# Strengthening of Reinforced Concrete Beams Subjected to Concentrated Loads Using Externally Bonded Fiber Composite Materials

**DOI:** 10.3390/ma15062328

**Published:** 2022-03-21

**Authors:** Paolo Foraboschi

**Affiliations:** Dipartimento di Culture del Progetto, Università IUAV di Venezia, 30135 Venice, Italy; paofor@iuav.it

**Keywords:** building remodeling, concentrated loads, external web reinforcement, shear capacity, side-bonded reinforcement, vertical concrete cantilever, vertical cracks

## Abstract

Renovation, restoration, remodeling, refurbishment, and the retrofitting of buildings often imply applying forces (i.e., concentrated loads) to beams that before were subjected to distributed loads only. In the case of reinforced concrete structures, the new condition causes a beam to bear a concentrated load with the crack pattern that resulted from the distributed loads which had acted before. If the concentrated load is applied at or near the beam’s midspan, the new shear demand reaches the maximum where cracks are vertical or quasi-vertical, and where inclined bars are not common according to any standards. So, the actual shear capacity can be substantially lower than new shear demand due to the concentrated load. This paper focuses on reinforced concrete beams whose load distribution has to be changed from distributed to concentrated and presents a design method to bring the beam’s shear capacity up to the new demand. The method consists of applying fiber composites (fiber-reinforced polymers or fiber-reinforced cementitious material) with fibers at an angle of 45° bonded to the beam’s web. This kind of external reinforcement arrangement has to comply with some practical measures, which are presented as well. The paper also provides the analytical model that predicts the concentrated load-carrying capacity of a beam in the strengthened state. The model accounts for the crack’s verticality, which nullifies the contributions of steel stirrups, aggregate interlock, and dowel action, and for the effective bond length of each fiber, which depends on the distance between the ends of the fiber and the crack it crosses.

## 1. Introduction

There is a time in their service life at which constructions no longer meet modern architectural demand. At that time, the construction may be subjected to renovation, restoration, remodeling, refurbishment, or retrofitting in order to meet modern demand (apart from monumental buildings).

Those activities may include placing new columns, walls, beams, or trusses into the building. Such new structural elements may be made supported by existing beams that previously carried floor slabs, which implies changing the load condition of a beam from distributed to concentrated. Moreover, the new concentrated load may be applied at or near the midspan of the existing beam.

If the beam is made of reinforced concrete (RC), the new load condition may be demanding even if the new concentrated load is lower than the resultant force of the previous distributed load. The reason lies in the crack pattern around the midspan, which is composed of stabilized vertical or quasi-vertical cracks, given that they were generated by the distributed loads that acted on the beam. That crack pattern implies a weakness, which often entails strengthening the beam.

This paper is devoted to those RC beams and presents a design method to increase the concentrated load-carrying capacity, including the relevant analytical model that predict the concentrated load-carrying capacity of RC beams both in the strengthened and unstrengthened condition.

## 2. Statement of the Issue Being Addressed

This section is devoted to framing the topic this paper deals with and to presenting the study’s statement of purpose, to which all elements of the paper relate logically.

### 2.1. Starting Knowledge

A lot of research work has been accomplished since the end of the nineteenth century on the shear behavior of RC structures, and outstanding papers are available in the literature.

An essential advance in knowledge was the well-known analogy between the shear strength of a web-reinforced concrete beam and a flat truss, postulated by Ritter (1899) [1], disseminated by Mörsch in Europe (1902) [2,3,4], and introduced by Withey into the American literature (1907) [5].

Another significant advance in knowledge was the strut-and-tie modeling, which divides a structure in B-regions (“B” standing for either beam or Bernoulli) and D-regions (“D” standing for either discontinuity or disturbed). The latter ones are in the vicinity of loads or geometric discontinuities, whereas the former ones are between the latter. Only the analysis and design of B-regions can proceed on a sectional basis for which plane sections remain plane, whereas the analysis and design of D-regions must proceed on a regional basis. Some of the many references are [6,7,8,9,10,11,12,13,14,15,16,17,18,19,20,21,22,23,24,25,26,27].

Then, a lot of research activity has been devoted to studying beam action and arch action. Some of the many references are [28,29,30,31,32,33,34,35,36,37,38,39,40,41,42,43,44].

### 2.2. Flat Truss Analogy: Review

The principal theoretical shear resisting system of an RC structure is an ideal parallel chord truss made of concrete and steel. The truss is composed of a top compression chord (the concrete from the neutral axis to the compression beam’s upper face, and the steel longitudinal reinforcement included into that region), a bottom tension chord (the steel longitudinal flexural reinforcement at the lower face of the beam), vertical tension ties (closed steel stirrups that span from the top chord to the bottom chord, sometimes with the addition of bent-up longitudinal steel bars, and/or inclined steel bars), and 45° inclined compression struts (the concrete between the shear cracks).

The joints of the members that compose the truss are assumed to be hinges. Accordingly, that system is called ‘pin-joined truss’. The pin-joined truss is statically determinate and stable. Consequently, the axial forces in the truss’s components can be calculated using the equilibrium only.

The truss mechanism exists only after the formation of the shear cracks, which cause the diagonal tension stresses to disappear.

### 2.3. Typical Cracking Pattern of Existing RC Beams

This point merely reviews some well-known generalities about the cracking behavior of a simply supported RC beam with web-reinforcement, under uniform loading [15,18,19,26,28,37,40,43]. The review includes extensions to the doubly fixed RC beam.

A uniform load induces a two-dimensional state of stress in the beam, whose maximum principal stress causes the concrete to crack if it exceeds the concrete tensile strength.

At midspan, the principal stresses are dictated by the bending moment only (which is true for the doubly fixed RC beam as well). Therefore, the principal stresses act on vertical planes and reach their maximum at the bottom of the cross-section. As a consequence, a crack at midspan initiates at the bottom face of the beam and propagates upwards vertically, up to the new compression zone in the upper part of the cross-section (flexural crack).

From the midspan to the supports, the interaction between bending moment and shear force increases. However, cracks are still initiated by bending moment, unless the beam is particularly deep. Each crack hence starts from the bottom face of the beam and propagates upwards. Nonetheless, the shear force is not nil, so propagation is driven by both bending and shear. That combined action causes cracks to deviate from verticality (flexural-shear cracks). Deviation increases from around the midspan, where cracks are quasi-vertical, to around the supports, where cracks are approximately at 45°.

At a support of a simply supported RC beam, the principal stresses are dictated by the shear force. Thus, the principal stresses act on planes at 45° and reach their maximum at the centroid. A crack due to shear force hence initiates at the centroid and propagates inwards at 45°, reaching the bottom edge of the beam (shear crack). 

Ultimately, the crack pattern of a simply supported RC beam exhibits vertical cracks at midspan, quasi-vertical cracks around the midspan, and progressively more splayed cracks toward the supports.

At and near the supports of a doubly fixed RC beam, there is some interaction between shear and bending. As a result, cracks can be either vertical (flexural cracks that initiate at the top edge) or at 45° (shear cracks). 

There is, however, a much lower chance of cracks that initiate at the centroid of the cross-section (shear cracks) appearing around the supports, whatever the restraints.

### 2.4. Typical Steel Web Reinforcement around the Midspan

The intended structural use of the vast majority of beams is to bear uniformly distributed loads, which requires that the shear force around midspan is small or nil. Accordingly, web reinforcement around the midspan of the vast majority of RC beams consists of relatively widely spaced steel stirrups, while neither bent-up longitudinal bars nor inclined tension ties are present around the midspan (no standards prescribe them), unless the design loads include concentrated loads.

The existing RC beams that this paper deals with were designed to bear uniform loads. So, the web reinforcement around the midspan has the above-described arrangement.

### 2.5. Shear Capacity of the Beam around the Midspan

If a concrete strut is intersected by a substantial crack, it does not transmit any significant compression force, since the concrete strut cannot close the crack that it intersects. This behavior implies that the ideal pin-joined truss is unstable.

Ergo, the classical analogy, whose concrete struts are at 45°, is valid only around the supports, but it does not hold true at the beam’s midspan. 

Given that a strut cannot be intersected by a substantial crack, each strut has to coincide with the concrete between two consecutive cracks—namely, the inclination of struts cannot be chosen arbitrarily but is dictated by the crack pattern. Accordingly, away from a beam’s support, the classical pin-joined truss analogy (whose concrete struts are at 45°) must be replaced by a pin-joined truss whose concrete struts are progressively less splayed from a support towards the midspan. Namely, the inclination of the struts must increase from 45° to 90° from a support to the midspan. 

Such a pin-joined truss extends the validity of the analogy to a greater fraction of the beam’s span. However, at and around the midspan, even that pin-joined truss loses its validity. Around the midspan, indeed, this concrete strut is vertical, so it has the same inclination (and the same position too) as a stirrup (as a vertical tie). Ergo, around the midspan, even this pin-joined truss is unstable. 

Ultimately, neither the classical pin-joined truss analogy nor the modified pin-joined truss analogy justifies any shear capacity of the beam’s segment where cracks are vertical. 

Nevertheless, the pin-joined truss analogy is valid at and around the supports, where the shear capacity plays a substantial role in the structural capacity, and it loses validity only where the shear demand of distributed loads is small. So, that deficiency plays a substantial role only in the specific case that this paper is devoted to.

Neither the fact that the pin-joined truss analogy does not hold true, nor the absence of web reinforcement automatically entails that an RC beam fails by shear [9,11,12,13,22,29,30,31,32,33,39,44]. It depends on which internal action will initiate cracking.

In the case of RC beams without web reinforcement, if cracking is initiated by shear force, the crack propagates inclined along the entire height, up to severing the beam. Ergo, without web reinforcement a shear crack causes the RC beam to fail—namely, the beam cannot bear the load that has cracked the concrete. 

Conversely, if cracking is initiated by the bending moment, the crack propagates either vertically or inclined along the section, up to stabilization (as long as the amount of tension reinforcement is adequate). Ergo, even without web reinforcement, flexural cracks and flexural-shear cracks allow a stabilized crack pattern to be reached—namely, the beam can bear the load that has cracked the concrete.

The shear resistant mechanisms of an RC beam with flexural and flexural-shear cracks without web reinforcement are arch action and beam action [28,29,30,31,32,33,34,35,36,37,38,39,40,41,42,43,44]. 

Arch action consists of an ideal concrete arch, given by the internal principal compression stresses. The shear capacity derives from the inclination of the internal compression, which is substantial at the supports but is negligible around the midspan (at midspan, compression stresses are horizontal). So, arch action provides the segment around the midspan with marginal shear capacity.

Beam action triggers when the cracks divide the tension zone into blocks, each of which consists of the concrete between two consecutive cracks. A block is usually referred to as ‘concrete cantilever’, since it acts as a cantilever with the base at the compression zone of the concrete and the free end in the concrete cover, and with the bond force variation applied at the free end.

Beam action mechanism can transfer the transverse shear force by means of the uncracked part of a cracked cross-section and can transfer the bond force variation between two consecutive cracks by means of the concrete cantilever.

In more detail, a flexural or flexural-shear cracked section of an RC beam without web reinforcement resists the transverse shear force by means of tangential stresses acting on the uncracked concrete, boosted by dowel action and aggregate interlock. Thus, the shear force is equilibrated by shear stresses on the transverse sections of the uncracked concrete, on the faces of the crack, and in the longitudinal tension reinforcement that crosses the crack. 

However, the tangential stresses acting on the uncracked concrete meet their limit when the principal tension stress reaches concrete tensile strength, which entails that this failure mode is brittle. At failure, consequently, the transverse relative displacements between the crack faces are small. Therefore, dowel action and aggregate interlock boost no more than moderately the shear transfer across the uncracked concrete zone of a cracked cross-section.

An RC beam without web reinforcement resists the bond force variation between two consecutive flexural or flexural-shear cracks by means of the bending, axial, and shear strength of the concrete cantilever, boosted by dowel action and aggregate interlock. Thus, the bond force variation is equilibrated by normal and shear stresses on the transverse sections of the concrete cantilever, and by shear stresses on the crack faces of the two adjacent cracks and in the length of the longitudinal tension reinforcement that cross those cracks.

The concrete cantilever meets its limit when the principal tension stress reaches concrete flexural strength, which requires that this failure mode is relatively ductile (i.e., not brittle, since it is dictated by bending, although not even ductile). However, the greater the angle of the cantilever to the beam’s axis, the lower the transverse relative displacements between the faces of the crack and, in turn, the lower the contributions of aggregate interlock and dowel action.

At failure, consequently, shear stresses transferred across flexural cracks due to aggregate interlock are no more than marginal, and dowel forces transferred across the cracks due to flexural stiffness of longitudinal reinforcement are negligible, whereas across flexural-shear cracks, those stresses are large, and those forces are significant.

In an RC beam with web reinforcement (with steel stirrups), beam action still takes place and acts in the same way as in an RC beam without web reinforcement, but more intensely, as stirrups make crack widths smaller, which boosts aggregate interlock, and make the concrete cover stronger, which boosts dowel action. In particular, beam action not only takes place for shear cracks too (it is of note that, in an RC beam with web reinforcement, shear cracks may exist), but also, aggregate interlock and dowel action provide the cantilever with even greater contributions than for flexural-shear cracks due to the lower crack’s inclination angle to the axis.

However, for flexural cracks of RC beams with stirrups the contributions of aggregate interlock and dowel action are negligible, as in the case of beams without stirrups.

Ultimately, around the midspan, where cracks are vertical (flexural cracks), the transverse relative displacements of the faces of a crack are negligible, and consequently, the contributions of aggregate interlock and dowel action are negligible as well, independently of the stirrups.

The conclusion that can be drawn relevant to the issue being addressed in this paper is that shear strength around the midspan of an RC beam derives only from the uncracked part of the cracked cross-sections, and from the concrete cantilevers (while the role played by aggregate interlock and dowel action is always marginal around the midspan). As a result, shear strength around the midspan of an RC beam is small. However, a concentrated load that is applied around the midspan induces the maximum shear force in that zone.

### 2.6. Gap Statement and Research Problem

According to the conclusion of Section 2.5, changing the load distribution of an RC beam from distributed along the span to concentrated around the midspan often requires increasing the shear strength of the beam.

Increasing the shear strength of a beam’s segment with vertical cracks requires referring to a mechanism different than the pin-joined truss. In fact, neither the classical analogy nor the truss model with variable strut inclination is able to predict shear strength where cracks are vertical. Moreover, not even aggregate interlock and dowel action mechanisms can be referred to, as they provide no more than marginal contributions where cracks are vertical.

Unfortunately, shear strengthening techniques that can be borrowed from the literature are based on the pin-joined truss and/or aggregate interlock and dowel action. Such strengthening techniques and the relevant models do not suit shear strengthening around the midspan, where cracks are vertical or quasi-vertical. That is the gap in knowledge that this paper aims to fill.

### 2.7. Study’s Statement of Purpose

The focus of the present research activity was to define a method for increasing the concentrated load-carrying capacity of RC beams, including the analytical model that predicts the capacity in the strengthened state.

The RC beams considered here are existing. So, they are cracked, and cracking is stabilized. Moreover, they do not have any reinforcement purposely designed for resisting concentrated loads around the midspan (since this was not the original intended use of those beams). 

**Figure 1:**
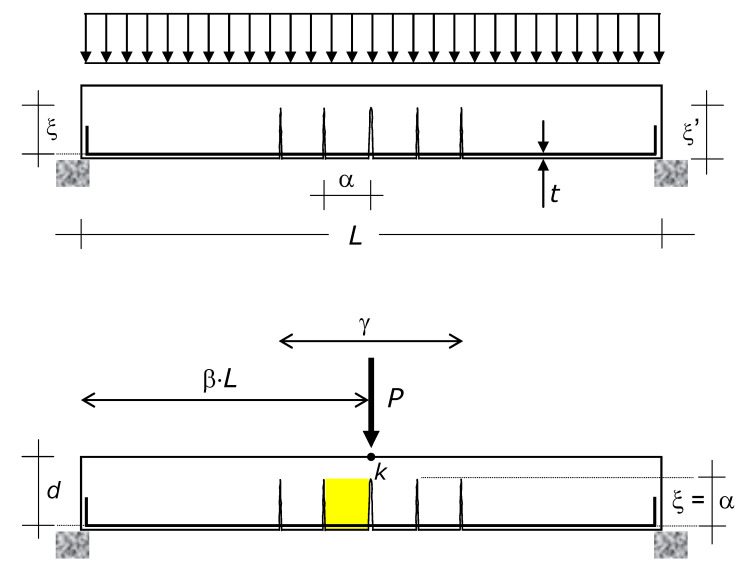
RC beam with vertical cracks around the midspan, which are spread along the length γ at spacing α. The upper diagram shows the loading condition that acted in the past and cracked the beam. The lower diagram shows the unstrengthened beam subjected to the new loading condition, i.e., the force *P* at point *k*, whose abscissa is β *L* (the beam could not bear that force). The figure also shows the span *L*, the depth *t* of the concrete cover, the crack depth ξ’, and the effective crack depth ξ. Failure is dictated by the shadowed cantilever.

The content and results of this paper, including the analytical model, apply to every RC beam, except for the beams embedded in the floor slab (i.e., this paper does not consider beams with thickness equal to the thickness of the floor slab they support). The reference beam, which is introduced in Section 4, is shown in Figure 1, which also shows the preceding and the new loadings, including the effect of the former, which creates the initial condition of the latter.

## 3. Shear Strengthening of Existing RC Beams

The main traditional shear strengthening techniques for RC beams are steel plate bonding (external plates connected to the beam with bolts, screws, or pins, or else epoxy-bonded), additional steel bars (external post-tensioning or internal bars embedded into the concrete and bonded with cement mortar or epoxy resin), and section enlargement. However, those techniques are complex to install and expensive (steel also include maintenance cost regarding corrosion). Moreover, they may be heavy and aesthetically unpleasant [45,46,47,48,49]. 

An innovation of the nineties of the past century was externally bonded fiber composite systems [50,51,52,53,54,55,56,57,58,59,60,61,62,63,64,65,66,67,68,69]. They consist of fibers embedded into either organic matrix (Fiber-Reinforced Polymers—FRP) or inorganic matrix (Fiber-Reinforced Cementitious Matrix—FRCM).

FRP and FRCM systems for strengthening RC structures are an excellent alternative to the above-mentioned traditional strengthening techniques, as the former offer substantial advantages over the latter: FRP and FRCM systems are easy to install, lightweight, very thin, noncorroding, and relatively economical.

In the case of FRP systems, supplemental externally bonded reinforcement for shear strengthening consists of either continuous textile fabric sheets or discontinuous fiber strips, whereas in the case of FRCM systems, it consists of continuous textile fabric sheets [70,71,72].

The configuration that reproduces the stirrups would consist of strips or sheets totally wrapped to the concrete section (fibers that envelop the cross-section). However, the cross-section (T-beam) or the floor slab (rectangular beam) implies that wrapping the beam with closed external reinforcement require breaking the flange or the floor slab, which is particularly invasive and expensive. Actually, wrapping the entire cross-section with the FRP or FRCM jacket is much less convenient than inserting new bars into the concrete, totally embedded from the top face to the bottom face of the beam, with the inclination that is wanted.

It follows that the typical shear strengthening configuration is the U-shaped reinforcement with the ends at the bottom of the beam’s flange or of the floor slab. Accordingly, shear strengthening of RC beams with external reinforcement is typically obtained by bonding either a continuous sheet with vertical fibers or spaced vertical strips onto the web (lateral sides) of the beam.

Unfortunately, U-shaped reinforcement cannot act as the web tension member of the pin-joined truss, since the external reinforcement does not reach the compression zone (top chord) or is not sufficiently anchored to the top chord. Namely, U-shaped reinforcement does not give rise to the vertical tension ties.

### Reinforced Concrete Beams Having Inadequate Shear Capacity

The topic of beams whose shear capacity is not enough or without web reinforcement needs in-depth analysis in order to frame the issues of beam’s shear strengthening around the supports (this section) and of midspan’s shear strengthening (next section). Although the first issue is well-known, the latter one is part of the gap that this paper aims to fill.

It is barely the case to observe that, if shear capacity is not enough, the beam with web reinforcement behaves as the beam without web reinforcement, since the stirrups have already provided the maximum force.

The variation of bending moment along an RC beam implies two internal forces, namely: (1) the transverse shear force across a cracked cross-section (across the concrete compression zone and crack), which is denoted by *V*, (2) and the bond force variation between two consecutive cracks (increase in tension force along the longitudinal reinforcement), which is denoted by Δ*T_s_*.

According to Section 2, those forces are resisted (1) by the uncracked concrete, i.e., concrete compression zone, (2) and the concrete cantilever, i.e., its built-in transverse section. The first of those two resisting systems is kind of boosted by dowel action and aggregate interlock, whereas the second one is substantially boosted by dowel action and aggregate interlock (splayed cracks).

(1) The maximum (ultimate) *V* that a cracked cross-section can bear is dictated by concrete tensile strength. When the maximum principal stress in the compression zone (maximum tension stress on an oblique plane, whereas compression stresses are on the plane at 90°) reaches concrete tensile strength, the cross-section reaches the maximum *V* that it can bear. A greater *V* initiates an oblique crack, which propagates inwards up to reaching the top of the beam in a direction, and the apex of the crack in the other direction and results in the severing of the beam into two parts.

(2) The maximum (ultimate) Δ*T_s_* that a concrete cantilever can bear is dictated by the bending strength, since the shear strength of the cantilever is almost always greater (although a cantilever is not slender). When the maximum principal stress at the built-in section of the concrete cantilever reaches concrete flexural strength, the cantilever reaches the maximum Δ*T_s_* that it can bear. A greater Δ*T_s_* breaks the built-in transverse section of the concrete cantilever, which causes the cantilever to fail and the beam to collapse by horizontal sliding of the lower part with respect to the upper part.

The lowest value of the load that makes the beam reach either the maximum *V* or the maximum (ultimate) Δ*T_s_* that the beam can bear is the ultimate load.

Generally speaking, hence, increasing the shear capacity of an RC beam requires increasing the maximum *V* and/or the maximum Δ*T_s_* that the RC beam can bear.

Using external reinforcement, an increase in the maximum *V* that a cracked cross-section can bear is obtainable only by wrapping the external reinforcement around the entire cross-section, which is an inconvenient technique, as previously observed. Actually, no U-shaped reinforcement allows the maximum tolerable *V* to be increased, since the uncracked concrete (compression zone) lies entirely, or almost entirely, in the flange, whereas the external reinforcement is bonded only on the web (usually, the ends of the reinforcement are at the bottom of the flange or of the floor slab).

Ultimately, no external reinforcement allows the maximum transverse shear force *V* that a cracked cross-section can bear to be increased. Namely, external reinforcement does not work at the support, let alone around the midspan.

Nevertheless, in the vast majority of RC beams, the load that makes the beam reach the maximum *V* is greater than the load that makes the beam reach the maximum Δ*T_s_* that can be carried by the RC beam.

Ultimately, shear strength is usually dictated by the bond force variation Δ*T_s_* and not by the transverse shear *V*.

U-shaped external reinforcement can increase the maximum bond force variation Δ*T_s_* that a cantilever can bear, since the maximum Δ*T_s_* occurs at the support and the fibers cross the cracks at the support with an angle different than zero.

In conclusion, typical shear strengthening techniques with externally bonded reinforcement—either sheets with vertical fibers or vertical strips bonded on the web (U-jacket configuration)—allow the maximum bond force variation Δ*T_s_* that a cantilever can bear to be increased, which, in turn, allows the shear strength of an RC beam to be increased.

## 4. Shear Strengthening of RC Beams around the Midspan

The conclusion of Section 3 is valid if and only if the angle between fibers and cracks is different than zero. 

**Figure 2:**
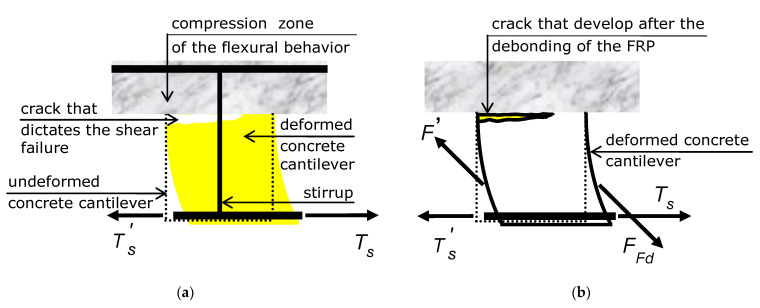
Concrete cantilever that dictates the capacity of the RC beam. (**a**): beam without external reinforcement. (**b**): beam with side-bonded FRP reinforcement, whose fibers are at ±45° (the sign of the angle depends on which of the two sides of the web the fibers are bonded to).

U-shaped reinforcement is vertical, so around the midspan, that angle is zero, since cracks are vertical too. Thus, U-shaped reinforcement does not provide any increase in shear strength around the midspan, just as a steel stirrup does not, and for the same reason (Figure 2a).

For the sake of completeness, U-shaped external reinforcement (or steel stirrups) would act as longitudinal reinforcement bonded onto (or embedded into) the concrete cantilever. Nevertheless, that behavior strengthens the built-in end section of the cantilever no more than marginally, since the cantilever’s cracking moment is greater than the cantilever’s plastic moment (unless the amount of external reinforcement or steel is huge). Thus, that contribution can be ignored.

Ultimately, no typical U-shaped reinforcements—neither sheets with vertical fibers nor vertical strips—allow the maximum bond force variation Δ*T_s_* tolerable by a vertical cantilever to be increased. 

The picture depicted above has proven that, in order to increase the maximum force variation Δ*T_s_* that a vertical cantilever can bear, the reinforcement has to provide the concrete cantilever with an oblique force. Ergo, the fibers of the web reinforcement have to be inclined (Figure 2b).

Analysis shows that the best fiber inclination is 45°. Not only does that angle optimize the behavior of the external reinforcement, but also, it makes bond application process easy.

A series of FRP strips at 45° would not produce the intended result, unless the spacing is unrealistically close. In fact, a diagonal strip could pass near the tip of the crack, which would imply applying a force with marginal or nil lever arm. In order to strength in shear the beam region with vertical cracks, thus, continuous textile fabric sheets with fibers at ±45° with respect to the beam axis must be used.

**Figure 3:**
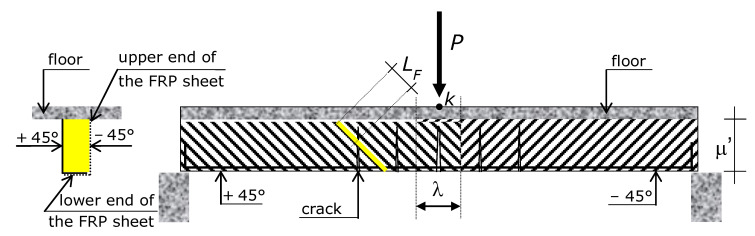
Externally bonded reinforcement that strengthens the RC beam subjected to a concentrated load: fabric sheets composed of unidirectional fibers at an angle of + 45° with the beam’s axis, where the shear force is positive, and at −45°, where the shear force is negative.

The sign of the fibers angle has to be that of shear internal action. Namely, from the left support to the application point of the concentrated load (point *K* of Figure 1), the fibers must be at +45°, whereas from the application point of the concentrated load to the right support, the fibers must be at −45°, where the positive angle is clockwise (Figure 3).

Reinforcement should be applied onto both the lateral faces of the beam, because if applied only onto one face, the section would be non-symmetric.

Fibers on one side of the web and fibers on the other side of the web cannot be continuous, i.e., a fiber attached onto one side of the web cannot be bent up and attached onto the other side. The reason is that fiber continuity would imply an angle of +45° (or −45°) at a lateral face and an erroneous angle of +45° (or −45°) at the opposite lateral face (Figure 3). In doing so, the external reinforcement on a lateral side would be effective, but that on the other lateral side would be ineffective. 

In other words, the angle of the fibers on a lateral side of a web must be opposite to the angle of the fibers on the opposite lateral sides of the web. Namely, fibers on the two sides are parallel. So, fibers cannot be continuous from one side to the opposite side of a web.

Ultimately, two different fabric sheets must be bonded onto the two lateral faces of the part of the beam on the left of the concentrated load, and other two different fabric sheets onto the part of the beam on the right, with fibers angle whose sign is opposite the sign of the opposite fibers.

At the cross-section where the concentrated load is applied (section *k*), the sheets overlap each other for the length λ > 2·0.707·*L_eff_*. The anchorage length (bond length) of an FRP sheet at the bottom face of the cross-section is λ’ > 2·0.707·*L_eff_*. At the bottom face, a sheet overlaps the sheet attached onto the other lateral face of the concrete section (unless the web is particularly wide). The figure also shows the anchorage length *L_f_* of a generic fiber.

Vertical cracks are due to bending moment, so vertical crack opening is driven by bending moment. Accordingly, the maximum width of a crack is at the bottom of the concrete section. So, the fibers at the bottom are subjected to the maximum elongation, which requires them being adequately anchored. In order to guarantee adequate bond length, thus, each sheet has to be bonded onto the lower face of the concrete section. Unless the web is particularly wide, hence, the two side-bonded reinforcements overlap each other onto the lower face of the concrete section. Moreover, if the web is relatively thin (less than 200–250 mm), a sheet has to be turned up onto the opposite lateral side (Figure 3).

The method proposed in this paper to increase the concentrated load-carrying capacity of RC beams is that described above. It is represented in Figure 3. The following sections are devoted to presenting the analytical model that predicts the concentrated load-carrying capacity of RC beams with the external reinforcement arrangement shown in Figure 3.

## 5. Basic Reference Structure

On one hand, the content of this paper is general. On the other hand, a reference structure saves the potential readers to invest too much effort in understanding the paper without losing generality.

The basic reference structure is the beam diagrammed in Figure 1. The span is denoted by *L*, the distance between the concrete extreme tension fiber (edge) and the center of the longitudinal bars (i.e., the concrete cover) by *t*, and the effective height by *d*.

The cross-section of the reference beam is constant along the span (prismatic beam), and the restraints are symmetric. The reference beam has either tee cross-section (T-beam) or rectangular section with depth greater than the depth of the floor slab it supports. Hence, the model directly includes not only the simply supported beam of Figure 1, but also doubly fixed beams. Nevertheless, the model can be easily extended to non-prismatic beams and non-symmetric restraints (fixed-roller beam).

The reference beam was subjected to a distributed load (upper diagram of Figure 1) and will be (or is) subjected to a concentrated load *P* (a force) applied at a point *K* around the midspan (for the sake of representativeness, Figure 1 places *K* at midspan). The position of point *K* is defined by β, i.e., the abscissa of *K* from the left support is β·L. The position of *K* is fixed, i.e., the new loading demand defines it (mobile load are not included).

The analytical model presented in the following sections predicts the maximum value of *P* that can be applied to the reference beam shown in Figure 1. That value is denoted by *P_ud_*, where the first subscript indicates that it is the ultimate concentrated load that the RC beam can bear without triggering the shear failure mode, and the second subscript indicates that it is the design concentrated load-carrying capacity of the RC beam. Namely, if the material parameters that are used apply the safety coefficients, *P_ud_* is the load to be used in the ultimate limit state verifications.

The beam is also subjected to a uniformly distributed load, denoted by *q*. The load *q* is produced by the beam’s own weight and some potential dead loads, so it is a minor load compared to *P*. Moreover, *q* would influence *P_ud_* marginal even if it were substantial, as proven in the following. For those reasons, *q* will be not included in modeling. Likewise, it is not included in the diagram of the reference structure. Nevertheless, equations can be easily modified in order to include *q*.

External reinforcement of the reference beam is an FRP material, but both the technique and the model presented in this paper also apply for FRCM materials.

According to Section 4, the RC beam is strengthened with side-bonded FRP sheets made of textile fabrics with fibers at +45° from the left support up to point *K*, and at −45° from the right support to point *K* (Figure 3).

The side-bonded reinforcement is composed of *N* layers. Hereinafter, a textile fabric sheet bonded onto one lateral face surface consists of one layer. Thus, the RC beam is strengthened by *N*/2 layers at one lateral face and *N*/2 layers at the other lateral face of the web. For example, a single-layer reinforcement bonded onto both the lateral faces calls for *N* = 2. 

The thickness of each layer of external reinforcement is denoted by *t_F_*, and the elastic modulus of the external reinforcement is denoted by *E_F_*. More specifically, *t_F_* is the fictitious thickness of a layer that, multiplied by the fictitious elastic modulus of the external reinforcement and by the real strain of the fibers, gives the force in the reinforcement per unit of width.

The total thickness of the external reinforcement bonded onto one lateral face is denoted by *t_F-tot_* (i.e., *t_F-tot_* = *t_F_* × *N*/2).

## 6. Assumptions of the Analytical Model and Further Nomenclature

The model derives the concentrated load-carrying capacity *P_ud_* from the equation of vertical translational equilibrium (i.e., Equation (1)). So, modeling is directed at predicting the resisting shear *V_ud_*, where the first subscript indicates that it is the ultimate (maximum) transverse shear force which a cross-section can bear without triggering the shear failure mode and the second subscript indicates that it is the design shear force of the RC beam (as long as design material strengths are used). Shear failure mode occurs by the bending failure of the shadowed concrete cantilever of Figure 1 (or by the symmetric one), in the strengthened condition (as detailed in the following, external fiber composite reinforcement reduces the bending moment induced by steel reinforcement).

Modeling uses the following further nomenclature: *M*_max_ = maximum bending moment in the beam due to *P_ud_* and *q* (whereas the role played by *q* in producing *M*_max_ is negligible compared to that of *P_ud_*), which occurs at the distance β·*L* from the support that provides the beam with the maximum vertical reaction (for a point load at midspan, as the representation of Figure 1: β = 0.5); ξ’ = crack depth; α = cracks spacing; and ξ = ξ’ − *t*, which is here referred to as effective crack depth (Figure 1).

Around the midspan, the fraction of internal shear action due to a uniformly distributed load is negligible with respect to the fraction due to *P_ud_*. Accordingly, *q* can be neglected in the vertical translational equilibrium equation:

(1) $$V_{ud}\;=\;\left(1\;-\;\beta \right)\;\cdot \;P_{ud}$$

On one hand, around the midspan, the bending moment due to *q* is maximum. On the other hand, however, *q* is a minor load, so *q·L* is negligible compared to *P_ud_*. Accordingly, the fraction of bending moment due to *q* can be neglected in comparison to that due to *P_ud_*:

(2) $$M_{\max}\;=\;\beta \cdot L\cdot V_{ud}\;=\;\beta \cdot L\cdot \left(1\;\;-\;\;\beta \right)\cdot P_{ud}$$

Equation (2) does not imply losing generality, since, in the marginal cases where the distributed load *q* is not minor, the equation can be easily modified so as to include the fraction of bending moment due to *q*.

Classical research about crack patterns of RC beams [19,23,35,73,74,75,76] supports the following two further assumptions (Figure 1).

(3) $$\alpha \;=\;\xi \;\equiv \;\xi^{\prime }\;-\;t$$

(4) ξ = 0.67⋅d

Combining assumptions (3) and (4) gives:

(5) $$\frac{\;d\;}{\alpha }\;=\;1.5$$

The last assumption that is made is about crack faces. Each face of a crack is assumed to be plane and to remain plane when loads make crack open, even when the two faces of a crack are tied (stitched, retained) by externally bonded reinforcement.

According to the above assumption, the crack profile is always triangular. This is a realistic assumption as well, since axial stiffness of fiber composite reinforcement is small, given that the thickness *t_F_* of each layer is very small.

## 7. Closed-Form Analytical Model

This section describes the mathematical development of the model, i.e., shows the equations and their derivation, providing explanation.

### 7.1. Force in the FRP Reinforcement Provisionally Neglecting Bond-Slip

**Figure 4:**
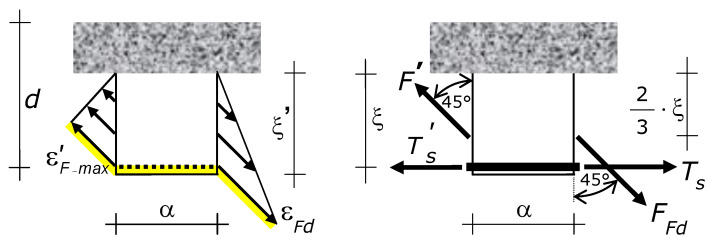
Concrete cantilever that fails (the midspan of the beam is on the right of the figure). On the left: strain profile in the external reinforcement at the two cracked sections that define the lateral sides (boundaries) of the concrete cantilever. The strain is linear, starting from zero at the neutral axis up to $\varepsilon_{\mathrm{Fd}}$ at the right crack and up to $\;\varepsilon_{F\text{-}\max}^{\prime }<\;\varepsilon_{\mathrm{Fd}}$ at the left crack. The external reinforcement bonded onto the concrete cover (which is shadowed) does not transmit any stress. On the right: forces transferred from the longitudinal steel bars (flexural reinforcement) and from the side-bonded reinforcement (the fibers are at 45°) to the concrete cantilever. The latter forces consist in a resisting contribution that makes the effects of the former forces less.

The side-bonded fiber composite reinforcement ties (sustain) the crack. The opening of a crack thus makes the fibers stretch (Figure 4). Fiber elongation produces a force. That force, which is denoted by *F*, has the direction of the fibers, i.e., ±45°.

Since strains in the fibers are induced by crack opening, the strain profile of the external reinforcement at a crack is equal to the crack opening profile. By virtue of the last mechanical assumption (Section 6), the strain profile of external reinforcement at a cracked section is triangular, and strains range from the maximum at the bottom (where the crack exhibits the maximum opening) to zero at the neutral axis (crack apex). The maximum strain, i.e., ε at the bottom of the crack, is denoted by $\varepsilon_{F}^{\max}$ (Figure 4).

The force *F* is produced by the triangular area of the above-described strain profile. In order to calculate *F*, two provisional assumptions are made, which will be removed in the following. The first is that there is no bond-slip between externally bonded reinforcement and concrete (not even at the bottom face, where stresses are maximum, which implies that the bottom anchorage length of the FRP reinforcement is adequate). As is well-known, ‘bond-slip’ is an oxymoronic term that means the relative movement of the reinforcement with respect to the surface it is attached onto or embedded into.

The second provisional assumption is that the external reinforcement coats the entire crack depth.

Under those provisional assumptions, *F* turns out to be:

(6) $$F\;=\;\frac{\varepsilon_{F}^{{}^{\max}}\;\cdot E_{F}\cdot \;t_{F}\;}{2}\cdot \xi^{\prime }\cdot N\;=\;\frac{\;\varepsilon_{F}^{{}^{\max}}\;\cdot E_{F}\cdot \;t_{F}\;}{2}\cdot \left(\xi \;+\;t\right)\cdot N$$

**Figure 5:**
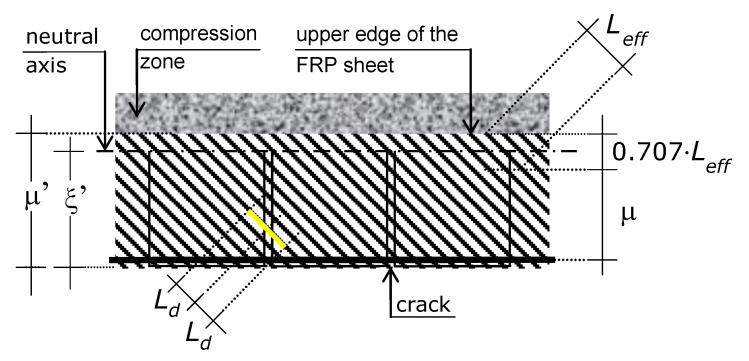
Fraction μ of μ’ that represents the effective height of the side-bonded fabric sheet, whereas the remaining part of μ’ is the anchorage (bond) length. The shadowed length *L_d_* + *L_d_* is the length of a fiber that crack opening causes to debond around the crack.

Owing to the two provisional assumptions that have been made, *F* provided by Equation (6) is the upper bound of the force, not the actual value. The reason why the actual value of *F* is lower is that there is some slip between the side-bonded reinforcement and the concrete lateral face it is attached onto (Figure 5). Thus, Equation (6) requires being refined in order to calculate the actual value of the force.

### 7.2. Bond-Slip and End-Debonding of the Fibers

The crack is tied (bridged) up to the loss of adhesion between side-bonded reinforcement and concrete (when proper installation is performed, failure occurs within the concrete in the form of removal of a layer of material, whose thickness may range from few millimeters to the whole concrete cover).

Failure can initiate at one of the ends of the side-bonded reinforcement or at the crack. According to [72], the former is called end debonding, whereas the latter intermediate debonding.

The strain that causes end debonding to occur depends on the bond length of the fiber. The greater the bond length, the greater the strain at which failure by end debonding occurs. However, end debonding strain can increase only to a certain limit. Any external reinforcement is characterized by an ‘optimal bond length’, which is the length, if exceeded, having no increase in the force transferred between external reinforcement and concrete. The optimal bond length is here denoted by *L_eff_* (it is also the effective bond length).

The maximum strain that a given external reinforcement can reach when bonded to a given surface is herein called ‘full end-debonding strain’ and is denoted by ε_Fd_. 

Both *L_eff_* and ε_Fd_ can be borrowed from the literature, in particular by codes [70,71,72]. This model uses the following expressions [72]:

(7) $$L_{eff}\;=\;0.47\cdot \sqrt[{2}]{\;\frac{\;E_{F}\cdot t_{F\text{-}tot}}{\;f_{ctd}\;}\;}\;[mm]$$

(8) $$\;\varepsilon_{\mathrm{Fd}}\;=\;0.35\cdot \frac{\sqrt[{4}]{f_{cd}\cdot f_{ctd}}}{\;\sqrt[{2}]{\;E_{F}\cdot t_{F\text{-}tot}\;}\;}$$

in which *f_ctd_* is the design value of the concrete cylinder tensile strength, and *f_cd_* is the design value of the concrete cylinder compressive strength.

Equations (7) and (8) do not incorporate *E_F_* and *t_F-tot_* individually, but their product. On one hand, *E_F_* and *t_F-tot_* (as well as *t_F_*) are hard to define and difficult to measure individually. Actually, they are ideal quantities borrowed from continuum mechanics, whereas fibers and textile fabrics are discontinuous. So, those quantities are fictitious. On the other hand, however, their product is the axial stiffness of the reinforcement, which is a quantity clearly defined and easy to be measured.

Equations (7) and (8) are written in a form that allows them to be used for collapse analysis, since the material strength values plugged into those equations (can) include partial factors for material properties to be used in the ultimate limit state verifications, as indicated by the subscript ‘d’.

Due to bond-slip, the shorter the actual bond length with respect to *L_eff_*, the lower the maximum strain that the external reinforcement can reach with respect to ε_Fd_.

Each fiber of the external reinforcement spans diagonally across the lateral side of the beam’s web (Figure 3) and continues at least onto the beam’s bottom face (lower side). More specifically, the upper end of a fiber is usually placed at the bottom of the flange (or, for a beam different than the reference structure, at the bottom of the floor slab), and the lower end is placed either on the bottom face of the beam or on the opposite lateral face.

Ultimately, every fiber ranges from the bottom face of the flange to the bottom side of the beam (or to the opposite lateral side of the beam).

Each fiber of the FRP reinforcement crosses a crack (infrequently two cracks). A fiber is hence anchored at the two sides of a crack, at a certain distance from the crack.

As explained at the beginning of this subsection, the force that a fiber provides a crack with also depends on bond-slip, which, in turn, depends on the length of anchorage of the fiber (bond length). The bond length of a fiber is equal to the distances between the end of the fiber and the intersection between fiber and crack. However, a fiber has one anchorage at each side of the crack, so it has two anchorages and then two bond lengths. In general, the two bond lengths are different from one another. The shortest of those two bond lengths, which is denoted by *L_F_*, is binding for the end-debonding. Consequently, *L_F_* dictates the force that the fiber can provide the crack with. Namely, if and how much the force is lower than the maximum value that can be transferred between external reinforcement and concrete depends on *L_F_* compared to *L_eff_*.

Ultimately, for *L_F_* ≥ *L_eff_*, where *L_eff_* is provided by Equation (7), fiber debonding strain can reach the maximum value that it can, i.e., ε_Fd_, for a given external reinforcement and bonding surface—namely, that provided by Equation (8)—whereas for *L_F_* < *L_eff_*, fiber debonding for strains is lower than ε_Fd_, and the extent of this depends on *L_eff_* − *L_F_*.

### 7.3. Maximum Force That the External Reinforcement Can Transmit to the Beam

If every fiber guaranteed *L_F_* ≥ *L_eff_*, Equation (6) would provide the force in the side-bonded reinforcement at a crack plugging ε_Fd_ for $\varepsilon_{F}^{\max}$. In other words, if the top and bottom edges of the side-bonded reinforcement are anchored for a length *L_F_* greater than *L_eff_*, the fibers can reach the full end-debonding strain, and *F* can be obtained using that resisting strain as maximum strain.

Obviously, the requirement *L_F_* ≥ *L_eff_* is more important at the bottom edge, where the strain is at maximum (moreover, stresses have the maximum lever arm).

For the bottom end of the external reinforcement, the requirement *L_F_* ≥ *L_eff_* is obtained by bonding each fabric sheet onto the bottom face of the concrete section, so that the two side-bonded reinforcements overlap each other, as detailed in Section 4. If the width of the web is lower than *L_eff_*, each fabric sheet has to be turned up onto the opposite lateral side.

For the top end of the external reinforcement, the requirement *L_F_* ≥ *L_eff_* cannot be guaranteed, since the flange (or the floor slab) often prevents the fabric sheet from being prolonged beyond the crack apex for a length equal to the anchorage (and sometimes even from reaching the apex). Thus, the model has to account for that behavior.

The effective bond length of the external reinforcement is different from the effective bond length of the fiber, because the latter is along the fiber, whereas the former is along the height of the web. Namely, due to the 45° angle of the fibers, the former is lower than the latter. More specifically, the effective bond length of the external reinforcement is equal to 0.707·*L_eff_*.

The side-bonded reinforcement coats the entire vertical crack only if:

μ’ − 0.707·*L_eff_* ≥ ξ’ (9)

where μ’ is the length of the concrete lateral face that the fabric sheet is bonded onto (Figure 3 and Figure 5).

If μ’ − 0.707·*L_eff_* < ξ’, at and near the crack apex, the fabric sheet has a bond length that does not allow the fibers to reach the full end-debonding strain, but only a fraction of it. In that case, the crack is not completely tied (stitched) by the side-bonded reinforcement. The greater the difference between the right term minus the left term of (9), the lower that fraction.

In that case, *F* is not produced by a triangular stress profile but by the area of the trapezoid inscribed into that triangle. The value of *F* is hence lower than that provided by Equation (6).

The value of μ’ depends on the depth of the flange (or of the floor slab). The effective depth μ of the side-bonded external reinforcement is therefore (Figure 3 and Figure 5) μ = μ’ − 0.707·*L_F_*.

The analytical model refers directly to μ = ξ’ and account for the actual value of μ indirectly, using a reduction factor η. This research work obtained η analytically. However, the analytical expression is cumbersome. All the more, η has a secondary weight on the results. With all that considered, a parametric analysis was carried out, so as to obtain approximate values of η. Those values are shown in Table 1. The values of η of Table 1 permit the real value of η to be replaced by an approximated value. It is of note that Table 1 assumes η = 1 for μ/ξ’ > 0.80, which is not preservative but introduces an irrelevant error.

The strengthening design should guarantee μ/ξ’ ≥ 0.20. Ratios lower than 0.20 should be avoided, because they would imply that only a minor part of the external reinforcement is well-bonded. 

Equation (6) can hence be replaced by:

(10) $$F\;=\;\frac{\varepsilon_{F}^{\max}\cdot E_{F}\cdot t_{F}}{2}\cdot \xi^{\prime }\cdot N\cdot \eta \;=\;\frac{\;\varepsilon_{F}^{\max}\cdot E_{F}\cdot t_{F}\;}{2}\cdot \left(\xi \;+\;t\right)\cdot N\cdot \eta$$

Bonding the external reinforcement onto the cracked concrete surface poses another bond problem, which needs solution—namely, intermediate debonding caused by flexural crack. A crack entails a discontinuity in the strain field of the concrete, which causes the reinforcement around a crack to detach. The point is whether and how much that detachment reduces *F*.

Let *L_d_* denote the length of side bonded reinforcement that has detached at each side of a crack (Figure 5). Only the fibers with *L_F_* ≥ *L_eff_* + *L_d_* can transfer the stress equal to *E_F_* × ε_Fd_ across a crack. However, if the bottom end of the external reinforcement guarantees that *L_F_* ≥ *L_eff_*, the entire side-bonded reinforcement guarantees that condition. Furthermore, only the fibers that are attached on the lateral side of the web for a length greater than *L_d_* can transfer some stress, whereas the fibers attached onto the lateral side of the web for a length lower than *L_d_* are loose.

Introduction of detachment around the crack into Equation (10) transforms that equation into (Figure 4 and Figure 5):

(11) $$F\;=\;\frac{\;\varepsilon_{F}^{{}^{\max}}\cdot E_{F}\cdot t_{F}\;}{2}\cdot \left(\xi \;+\;t\;-\;0.707{\cdot L}_{d}\right)\cdot N\cdot \eta$$

Observation has proven that 20 < *L_d_* < 80 mm [72]. On the realistic assumption that 0.70·*L_d_* = *t*, Equation (11) turns into:

(11a) $$F\;=\frac{\;\varepsilon_{F}^{{}^{\max}}\cdot E_{F}\cdot t_{F}\;}{2}\cdot \xi \cdot N\cdot \eta \;=\;0.333\cdot E_{F}\cdot t_{F}\cdot d\cdot N\cdot \eta \cdot \varepsilon_{F}^{\max}$$

The distance of the force *F* from the neutral axis is 2·ξ/3 and the distance of *F* from the steel reinforcement is ξ/3.

Let *F_Fd_* denote the maximum value that the force *F* can reach. The force *F_Fd_* is dictated by end debonding:

(12) $$\;F_{Fd}=\;\frac{\;\varepsilon_{\mathrm{Fd}}\cdot E_{F}\cdot t_{F}}{2}\cdot \xi \cdot \eta \cdot N\;=\;0.333\cdot d\cdot \varepsilon_{\mathrm{Fd}}\cdot E_{F}\cdot t_{F}\cdot d\cdot \eta \cdot \;N$$

The side-bonded reinforcement elongates in proportion to the crack width. So, ε_Fd_ and *F_Fd_* occurs at the crack that transmits the maximum bending moment.

### 7.4. Internal Actions in the Concrete Cantilever

**Figure 6:**
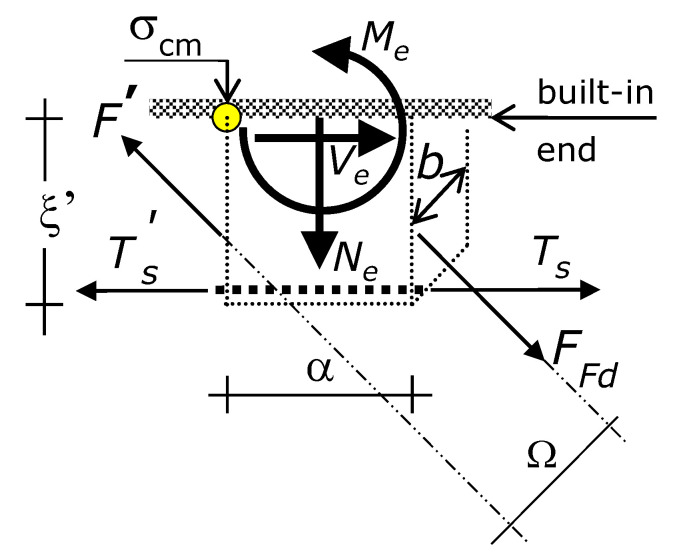
Forces applied to the concrete cantilever, internal actions, and maximum stress. The steel longitudinal bars and the side-bonded reinforcement induce the internal actions *M_e_*, *N_e_*, and *V_e_* at the built-in end of the concrete cantilever. At debonding, *M_e_* and *N_e_* induce the maximum tension stress σ_cm_, which is shown in the figure, including the point where it occurs (shadowed circle).

The longitudinal bond force variation Δ*T_s_* induces a bending moment Δ*T_s_*·ξ at the built-in end of the vertical concrete cantilever (Figure 4 and Figure 6). The global behavior of the beam is linear-elastic, because the steel reaches plasticity only for displacements greater than those at which debonding occurs. So, at failure the lever arm of the internal couple is 0.89·*d* (i.e., the distance between the center of the tension longitudinal steel and the center of the compression stresses is 0.89·*d*). 

Once the internal lever arm is fixed, the sole equilibrium furnishes the relation between Δ*T_s_* and the shear action *V* in the cracked section:

(13) $$0.89\cdot d\cdot \Delta \;T_{S}\;=\;V\cdot \alpha \;\to \;\Delta \;T_{S}\;=\;\frac{V\cdot \alpha }{\;0.89\cdot d\;}$$

The force *F* in the side-bonded reinforcement varies along the span, since it is in proportion to the width of the crack that it ties (Section 7.3). In turn, crack widths are in proportion to the bending moment along the span. Ergo, along the span, the force *F* varies in the same way as the force *T_s_* in the longitudinal steel reinforcement (the internal lever arm *d* is constant).

The above sequence of implications leads to the conclusion that *F* and *T_s_* reach their maximum at midspan, and that those forces decrease from the midspan to the supports. It follows that the variation of *F* at the two sides of the concrete cantilever induces a bending moment in the built-in end of the concrete cantilever that has the same sign of the bending moment induced by the variation of *T_s_*. This fact would seem to invalidate the proposed strengthening technique.

Nevertheless, the two forces *T_s_* at the two sides of the concrete cantilever (one at a crack and the other at the consecutive crack) are coaxial. Namely, the lever arm between those forces is zero. On the contrary, the two forces *F* at the two sides of the cantilever are inclined, so the lever arm between those forces is not zero.

Let Ω denote the lever arm between the two parallel forces *F* applied to a concrete cantilever, one force at the right side and the other force at the left side (Figure 6). By virtue of Ω, the two forces *F* induce a bending moment whose sign is opposite to the sign of the bending moment induced by the variation of *T_s_*, and by the variation of the modulus of *F* as well.

As a result, the side-bonded reinforcement makes the tension stresses in the concrete cantilever decrease, i.e., the externally bonded reinforcement induces stresses with opposite sign to stresses induced by the longitudinal steel reinforcement (compression the latter, tension the former).

Ultimately, the forces that increase the strength of the concrete cantilever are *F_Fd_* at the lateral side of the cantilever closer to the midspan and *F*’ at the lateral side more distant from the midspan (with *F*’ < *F_Fd_*). Tforce *F*’ will be defined in the following. The inclination of forces *F_Fd_* and *F* is +45° (Figure 2 and Figure 4).

The forces *F_Fd_* and *F*’ induce tension axial force, transverse shear force (i.e., shear transverse to the cantilever axis), and bending moment in the concrete cantilever. The section of the concrete cantilever that dictates failure is the built-in end (the section of the cantilever that is fixed to the compression zone of the beam).

At the built-in end, the internal actions due to the external reinforcement are the tension axial force *N_e_*, the transverse shear force *T_e_*, and bending moment *M_e_* given by the following expressions (Figure 6).

(14) $$N_{e}=\frac{\;\sqrt[{2}]{\;2\;}\;}{2}\cdot \left(F_{Fd}\;-\;F\prime \right)$$

(15) $$T_{e}=\frac{\;\sqrt[{2}]{\;2\;}\;}{2}\cdot \left(F_{Fd}\;-\;F^{\prime }\right)$$

(16) $$M_{e}=\frac{\;\sqrt[{2}]{\;2\;}\;}{3}\cdot \left(F_{Fd}\;-\;F^{\prime }\right)\cdot \xi \;-\;\frac{\;\sqrt[{2}]{\;2\;}\;}{4}\cdot \left(F_{Fd}\;-\;F^{\prime }\right)\cdot \alpha$$

This is to highlight that *T_e_* of Equation (15) and *M_e_* of Equation (16) are resisting internal actions—namely, they make tension stresses due to Δ*T_s_* lower. So, *T_e_* and *M_e_* belong to the resisting system. On the contrary, the tension axial force *N_e_* of Equation (14) belongs to the force system (and not to the resisting system), since this induces tension stresses in the concrete cantilever, as does Δ*T_s_*. In other words, with all the other parameters being the same, the greater *T_e_* and *M_e_*, the greater the increase in concentrated load-carrying capacity provided by the externally bonded reinforcement. On the contrary, the greater *N_e_*, the lower the increase in concentrated load-carrying capacity provided by the externally bonded reinforcement.

Ultimately, the internal actions in the vertical concrete cantilever are the shear force and bending moment due to the longitudinal bond force variation Δ*T_s_* and the axial force, shear force, and bending moment due to the external reinforcement. Eventually, the internal actions of Equations (14)–(16) allow the RC beam to carry greater concentrated loads around the midspan.

Equation (13) shows that the modulus of Δ*T_s_* is constant along the span, since *V*, α, and *d* are constant. The effects induced by Δ*T_s_* are thus the same in each vertical concrete cantilever. It follows that failure is dictated by the concrete cantilever where the effects induced by Equations (14)–(16) are minimum.

The bending moment of Equation (16) reaches its minimum where the two forces *F* acting on a cantilever are minimum, and vice versa. Since *F* is in proportion to crack width, and crack width is in proportion to the bending moment, that minimum occurs at the vertical (or quasi-vertical) concrete cantilever that is farthest from point *K* (in the case of the reference structure, that is farthest from the midspan), and vice versa.

The tension axial force of Equation (14) reaches its maximum where the two forces *F* acting on a cantilever are maximum, and vice versa. Again, *F* is in proportion to crack width, and crack width is in proportion to the bending moment, so that maximum occurs at the vertical (or quasi-vertical) concrete cantilever that is nearest point *K* (in the case of the reference structure, nearest the midspan), and vice versa.

The tension axial force *N_e_* is substantial. As a result, the combination between *N_e_* and the bending moment *M_e_* induces the lowest effect in the vertical cantilever where *N_e_* is at maximum, although in that cantilever, *M_e_* is at maximum too.

To sum up the above analysis, the minimum compression stress that reduces the maximum tension stress induced by Δ*T_s_* occurs in the vertical cantilever at point *K* or nearest point *K* (in the case of the reference structure, at midspan). Ergo, failure is dictated by the concrete cantilever at point *K* (or that is nearest point *K*). If the concentrated load is at midspan, as in the reference beam, failure is dictated by the concrete cantilever at midspan.

### 7.5. Mode of Failure of the RC Beam with Side-Bonded FRP Reinforcement

Debonding is a very brittle failure mode. The classical U-shaped external reinforcement used to strengthen in shear RC beams around the ends (at the beam’s supports, where shear demand due to distributed loads is maximum) debonds before dowel action and aggregate interlock have given substantial contributions. As a result, the actual increase in shear strength of the beam provided by the classical external reinforcement is moderate.

According to Section 2.5, dowel action and aggregate interlock are marginal around midspan (in fact, those effects are ignored herein). This reveals that the proposed externally bonded fiber composite reinforcement is always a viable technique to strengthen in shear RC beams around the midspan.

The possible failure modes of an RC beam with side-bonded reinforcement whose configuration is that described in Section 4 are i. cracking of the concrete cantilever ii. and debonding of the external reinforcement. i. Cracking of the concrete cantilever is dictated by concrete flexural strength, which implies that the concrete displays some plasticity (inelasticity in tension). ii. Debonding of the external reinforcement is dictated by concrete tensile strength (by mode II of fracture mechanics), which is totally brittle. Ergo, failure occurs by ii. debonding of the external reinforcement. 

Failure triggers because the side-bonded reinforcement stops providing the concrete cantilever with the forces *F_Fd_* and *F*’, which results in a reduction in the resisting internal actions of Equations (14)–(16) to zero, which, in turn, causes the cantilever to fail. In fact, without those resisting internal actions, the concrete cantilever cannot resist the bending moment that is induced by the steel reinforcement at its built-in end. Once the cantilever fails, the whole beam fails.

To sum up: 1. the capacity of carrying a concentrated load applied around the midspan is dictated by the debonding of the external reinforcement, since the concrete cantilever, although not slender, fails by bending moment, and moreover, it displays some plasticity. 2. Debonding occurs when the maximum strain in the external reinforcement, $\varepsilon_{F}^{\max}$, reaches the full end debonding strain, ε_Fd_. 3. To fail is the vertical concrete cantilever whose lateral side at (near) the midspan is a face of the crack where the external reinforcement exhibits $\varepsilon_{F}^{\max}$ = ε_Fd_. 4. Failure occurs at the built-in (horizontal) section of that cantilever. 5. Debonding causes that cantilever to lose the forces exchanged with the external reinforcement, and without those resisting forces, the cantilever cannot equilibrate the bending moment induced by the variation of longitudinal force in the steel bars (i.e., without the external reinforcement, the moment induced by Δ*T_s_* at the built-in end cannot be resisted). 6. When the cantilever fails, the beam collapses.

At debonding, the bending moment produced by Δ*T_s_* is resisted by two bending moments acting on the built-in section—namely, the resisting bending moments provided (a) by the forces in the side-bonded reinforcement, and (b) by the stresses acting on the built-in section of the cantilever.

(a) The resisting bending moment provided by the forces in the side-bonded reinforcement derives from *F_Fd_* and *F*’ and their lever arm, Ω. The former force is already known, i.e., it is given by Equation (12), whereas the latter force is calculated in the following. As previously detailed (Section 7.4), the resisting contribution produced by this bending moment is in some degree reduced by the tension axial force induced by *F_Fd_* and *F*’.

(b) The resisting bending moment provided by the stresses acting onto the built-in horizontal section of the cantilever requires knowing the flexural behavior of the concrete cantilever at debonding of external reinforcement.

Theoretical analysis carried out within this research activity demonstrates that at debonding, the maximum tension strain in the concrete cantilever is beyond the elastic limit, although always lower than the cracking tensile strain. Accordingly, the stress distribution on the concrete section is elasto-plastic.

Modeling assumes that stresses acting onto the built-in section at debonding have a bi-triangular profile, with maximum equal to the design value of the concrete cylinder tensile strength *f_ctd_*. That assumption is simultaneously realistic and conservative, because the bending moment given by the stresses that are ignored is small and would increase the strength.

### 7.6. Maximum Tension Stress in the Concrete Cantilever Induced by the Loads

Let σ_cm_ denote the maximum tension stress in the concrete cantilever. The stress σ_cm_ occurs at one edge of the built-in end of the cantilever (the edge more distant from the midspan), as shown by Figure 6. According to Section 7.5, that tension stress is induced by the bending moment due to steel reinforcement and fiber composite external reinforcement, and by the axial force due to fiber composite external reinforcement (Figure 6). On the contrary, the shear force in the cantilever does not provide any contribution to the normal stresses.

The bi-triangular stress profile, which implies a linear-elastic behavior of the concrete cantilever, requires that σ_cm_ is equal to:

(17) $$\begin{array}{l} \sigma_{\mathrm{cm}}\;=\;\frac{\;\xi \cdot \Delta T_{S}}{\;W\;}\;+\;\frac{\;\sqrt[{2}]{\;2\;}\;}{3\cdot W}\cdot \left(F_{Fd}\;-\;F^{\prime }\right)\cdot \xi \;+\;\\ \frac{\sqrt[{2}]{\;2\;}\;}{2\cdot b\cdot \alpha }\cdot \left(F_{Fd}\;-\;F^{\prime }\right)\;-\;\frac{\;\sqrt[{2}]{\;2\;}}{\;4\;}\cdot \left(F_{Fd}\;-\;F^{\prime }\right)\cdot \frac{\alpha }{W} \end{array}$$

where *W* is the section modulus of the built-in end of the concrete cantilever.

Plugging Equation (13) into Equation (17) leads to:

(18) $$\begin{array}{l} \sigma_{\mathrm{cm}}\;=\;\frac{V\cdot \alpha \cdot \xi }{\;0.89\cdot d\cdot W\;}\;+\;\frac{\;\sqrt[{2}]{\;2\;}\;}{3\cdot W}\cdot \left(F_{Fd}\;-\;F^{\prime }\right)\cdot \xi \;+\;\\ \frac{\sqrt[{2}]{\;2\;}\;}{2\cdot b\cdot \alpha }\cdot \left(F_{Fd}\;-\;F^{\prime }\right)\;-\;\frac{\;\sqrt[{2}]{\;2\;}}{\;4\;}\cdot \left(F_{Fd}\;-\;F^{\prime }\right)\cdot \frac{\alpha }{W} \end{array}$$

On substituting Equation (4) for α and ξ, Equation (18) becomes:

(19) $$\begin{array}{l} \sigma_{\mathrm{cm}}\;=\;\frac{\;0.444\cdot V\cdot d^{\;2}\;}{\;0.89\cdot d\cdot W\;}\;+\;\frac{\;\sqrt[{2}]{\;2\;}\cdot 0.667\;}{3\cdot W}\cdot \left(F_{Fd}\;-\;F^{\prime }\right)\cdot d\;+\;\\ \frac{\sqrt[{2}]{\;2\;}\cdot 1.5\;}{2\cdot b\cdot d}\cdot \left(F_{Fd}\;-\;F^{\prime }\right)\;-\;\frac{\;\sqrt[{2}]{\;2\;}\cdot 0.667\;}{\;4\;}\cdot \left(F_{Fd}\;-\;F^{\prime }\right)\cdot \frac{d}{\;W} \end{array}$$

Rearranging terms, Equation (19) can be expressed in the following form:

(20) $$\begin{array}{l} \sigma_{\mathrm{cm}}=\frac{\;0.499\cdot V\cdot d\;}{\;W\;}+\;\frac{\;0.314\;}{W}\cdot \left(F_{Fd}\;-\;F^{\prime }\right)\cdot d+\;\\ \frac{\;1.061\;}{b\cdot d}\cdot \left(F_{Fd}\;-\;F^{\prime }\right)\;-\;0.236\cdot \left(F_{Fd}\;-\;F^{\prime }\right)\cdot \frac{d}{\;W} \end{array}$$

### 7.7. Gradient of the Force in the Side-Bonded Reinforcement

According to Section 7.4, failure is dictated by the vertical concrete cantilever at point *K* or nearest point *K* (in the reference beam, failure is dictated by the concrete cantilever at midspan). Accordingly, the vertical face of the concrete cantilever at point *K* (at midspan) is subjected to the maximum bending moment *M*_max_, which causes the side-bonded reinforcement at that face to reach the full end debonding strain ε_Fd_.

The lateral face of that concrete cantilever more distant from point *K* (from the midspan) is subjected to a bending moment, which is denoted by *M*’. That bending moment derives from the variation of *M* between the two consecutive cracks that define the boundaries of the concrete cantilever. The moment *M*’ causes the external reinforcement to reach a maximum tension strain, which is denoted by $\varepsilon_{F\text{-}\max}^{\prime }$.

The first modeling assumption allows the following relationship to be established: *M*’ = *M*_max_ − α·*V_ud_*.

Given that *M*_max_, *M*’, and ε_Fd_ are known, the strain $\varepsilon_{F\text{-}\max}^{\prime }$ can be derived from a proportion:

(21) $$\varepsilon_{F\text{-}\max}^{\prime }\;=\;\varepsilon_{\mathrm{Fd}}\;\cdot \frac{M^{\prime }}{M_{\max}\;}$$

Modeling assumptions allow Equation (21) to be written in the following form:

(22) $$\begin{array}{l} \varepsilon_{F\text{-}\max}^{\prime }=\;\varepsilon_{\mathrm{Fd}}\cdot \frac{M_{\max}\;-\;\alpha \cdot V_{ud}}{M_{\max}}\;=\;\varepsilon_{\mathrm{Fd}}\cdot \left(1\;-\;\frac{\alpha \cdot V_{ud}\;}{M_{\max}}\;\right)\\ =\;\varepsilon_{\mathrm{Fd}}\cdot \left(1\;-\;\frac{\;0.667\cdot d\cdot V_{ud}}{M_{\max}}\;\right) \end{array}$$

Again, modeling assumptions allow Equation (22) to be rewritten:

(23) $$\varepsilon_{F\text{-}\max}^{\prime }\;=\;\varepsilon_{\mathrm{Fd}}\cdot \left(1\;-\;\frac{0.667\cdot d\cdot V_{ud}\;}{\beta \cdot L\cdot V_{ud}}\;\right)\;=\;\varepsilon_{\mathrm{Fd}}\cdot \left(1\;-\;\frac{\;0.667\cdot d\;}{\;\beta \cdot L\;}\;\right)$$

The force *F*’ in the external reinforcement at the side of the concrete cantilever more distant from the midspan derives from the triangular area of the strain profile whose maximum is $\varepsilon_{F\text{-}\max}^{\prime }$, given by Equation (23):

(24) $$\begin{array}{l} F^{\prime }=\;\frac{\varepsilon_{F\text{-}\max}^{\prime }\cdot E_{F}\cdot t_{F}}{2}\cdot \xi \cdot \eta \;\cdot N\\ =\;\frac{\;\varepsilon_{\mathrm{Fd}}\cdot E_{F}\cdot t_{F}\;}{2}\cdot 0.667\cdot d\cdot \eta \cdot N\;\cdot \left(1\;-\;\frac{\;0.667\cdot d\;}{\;\beta \cdot L\;}\;\right)\\ =\;\varepsilon_{\mathrm{Fd}}\cdot E_{F}\cdot t_{F}\cdot d\cdot \eta \cdot N\cdot \left(0.333\;-\;\frac{\;0.222\cdot d\;}{\;\beta \cdot L\;}\;\right) \end{array}$$

### 7.8. Shear Strength of the RC Beam in the Strengthened State

According to Section 7.5, the maximum tension stress σ_cm_ in the concrete cantilever at debonding of the external reinforcement is equal to the concrete tensile strength *f_ctd_*. Plugging *f_ctd_* into Equation (20):

(25) $$\begin{array}{l} \;f_{ctd}\;=\;\frac{\;0.499\cdot d\cdot V_{ud}\;}{\;W\;}\;+\;\frac{\;0.314\;}{W}\cdot \left(F_{Fd}\;-\;F^{\prime }\right)\cdot d+\;\\ \frac{\;1.061\;}{b\cdot d}\cdot \left(F_{Fd}\;-\;F^{\prime }\right)\;-0.236\cdot \left(F_{Fd}\;-\;F^{\prime }\right)\cdot \frac{d}{\;W} \end{array}$$

Equation (25) may be solved for *V_ud_*. In so doing, the ultimate shear force is found:

(26) $$\begin{array}{l} V_{ud}\;=\;\frac{\;2\cdot W\;}{d}\cdot [f_{ctd}\;-\;\frac{\;0.314\;}{W}\cdot \left(F_{Fd}\;-\;\;F^{\prime }\right)\cdot d\;\\ +0.236\cdot \left(F_{Fd}\;-\;\;F^{\prime }\right)\cdot \frac{d}{W}\;-\;\frac{\;1.061\;}{b\cdot d}\cdot \left(F_{Fd}\;-\;\;F^{\prime }\right)\;] \end{array}$$

Equation (26) may be expressed in the following form:

(27) $$\begin{array}{l} V_{ud}\;=\;\frac{\;2\cdot W\cdot f_{ctd}\;}{d}-\;0.629\cdot \left(F_{Fd}\;-\;F^{\prime }\right)\\ +\;0.471\cdot \left(F_{Fd}\;-\;F^{\prime }\right)\;-\;\frac{\;2.121\cdot W\;}{\;b\cdot d^{2}}\cdot \left(F_{Fd}\;-\;F^{\prime }\right) \end{array}$$

Modeling assumptions represented by Equations (3)–(5) allow the section modulus *W* to be expressed as a function of the geometric parameters. On substituting α into the formula of the section modulus *W* (rectangular section): *W* = 0.074·*b*·*d*^2^. On substituting the expression of *W* into Equation (27):

(28) $$\begin{array}{l} V_{ud}\;=\;\frac{\;0.148\cdot b\cdot d^{2}\cdot f_{ctd}\;}{d}\;-\;0.629\cdot \left(F_{Fd}\;-\;F^{\prime }\right)\\ +\;0.471\cdot \left(F_{Fd}\;-\;F^{\prime }\right)\;-\;\frac{\;0.157\cdot b\cdot d^{2}}{b\cdot d^{2}{}^{}}\cdot \left(F_{Fd}\;-\;F^{\prime }\right) \end{array}$$

Equation (27) can be rewritten as:

(29) $$\begin{array}{l} V_{ud}\;=\;0.148\cdot b\cdot d\cdot f_{ctd}-\;0.629\cdot \left(F_{Fd}\;-\;F^{\prime }\right)\\ +\;0.474\cdot \left(F_{Fd}\;-\;F^{\prime }\right)\;-\;0.157\cdot \left(F_{Fd}\;-\;F^{\prime }\right) \end{array}$$

The sum and the difference of the terms into the three round brackets can be obtained plugging Equations (12) and (24) into each round bracket. In so doing, the round bracket with the minus sign turns out to be:

(30) $$F_{Fd}\;-\;F^{\prime }=\;0.33\cdot d\cdot \varepsilon_{\mathrm{Fd}}\cdot E_{F}\cdot t_{F}\cdot d\cdot \eta \cdot \;N\;-\;\varepsilon_{\mathrm{Fd}}\cdot E_{F}\cdot t_{F}\cdot d\cdot \eta \cdot N\cdot \left(0.333\;-\;\frac{\;0.222\cdot d\;}{\;\beta \cdot L\;}\;\right)$$

Equation (30) can be simplified into:

(31) $$F_{Fd}\;-\;F^{\prime }\;=\;0.222\cdot \varepsilon_{\mathrm{Fd}}\cdot E_{F}\cdot t_{F}\cdot \eta \cdot N\;\cdot \;\frac{\;d^{2}\;}{\;\beta \cdot L\;}$$

In the same fashion, the round bracket with the plus sign turns out to be:

(32) $$F_{Fd}\;-\;F^{\prime }=\;0.333\cdot d\cdot \varepsilon_{\mathrm{Fd}}\cdot E_{F}\cdot t_{F}\cdot d\cdot \eta \cdot \;N\;+\;\varepsilon_{\mathrm{Fd}}\cdot E_{F}\cdot t_{F}\cdot d\cdot \eta \cdot N\cdot \left(0.333\;-\;\frac{0.222\cdot d}{\;\beta \cdot L\;}\right)$$

Equation (32) can be simplified into:

(33) $$F_{Fd}\;-\;F^{\prime }=\;\varepsilon_{\mathrm{Fd}}\cdot E_{F}\cdot t_{F}\cdot \eta \cdot \;N\cdot \left(0.666\cdot d\;-\;\frac{\;0.222\cdot d^{2}\;}{\;\beta \cdot L\;}\right)$$

Plugging Equations (31) and (33) into (29):

(34) $$\begin{array}{l} V_{ud}\;=\;0.148\cdot b\cdot d\cdot f_{ctd}-\;0.140\cdot \varepsilon_{\mathrm{Fd}}\cdot E_{F}\cdot t_{F}\cdot \eta \cdot N\;\cdot \;\frac{\;d^{2}\;}{\;\beta \cdot L\;}\\ +\;\varepsilon_{\mathrm{Fd}}\cdot E_{F}\cdot t_{F}\cdot \eta \cdot \;N\cdot \left(0.314\cdot d\;\;-\;\;\frac{\;\;0.105\cdot d^{2}\;}{\;\beta \cdot L\;}\right)\;-\\ 0.035\cdot \varepsilon_{\mathrm{Fd}}\cdot E_{F}\cdot t_{F}\cdot \eta \cdot N\;\cdot \;\frac{\;d^{2}\;}{\;\beta \cdot L\;} \end{array}$$

Equation (34) allows the ultimate design resisting shear, *V_ud_,* to be finally worked out:

(35) $$V_{ud}\;=\;0.148\cdot b\cdot d\cdot f_{ctd}\;+\;0.314\cdot \varepsilon_{\mathrm{Fd}}\cdot E_{F}\cdot t_{F}\cdot \eta \cdot \;N\cdot d\;-\;0.280\cdot \varepsilon_{\mathrm{Fd}}\cdot E_{F}\cdot t_{F}\cdot \eta \cdot N\;\cdot \;\frac{\;d^{2}}{\;\beta \cdot L\;}$$

Equation (35) permits the maximum concentrated load that the strengthened RC beam can carry to be derived. The final formula that predicts the concentrated load-carrying capacity of a strengthened RC beam, *P_ud_,* is:

(36) $$P_{ud}\;=\;\frac{V_{ud}\;}{1\;-\;\beta \;}$$

where *V_ud_* is given by Equation (35).

Ultimately, Equations (35) and (36) constitute the model that allows the RC beams strengthened in the fashion described in Section 4 to be analyzed and checked against concentrated loads. In fact, the two-equation model provides the concentrated load-carrying capacity to compare to the demand, which allows the ultimate limit state verifications to be performed.

## 8. Experimental Verification of the Theoretical Model

**Figure 7:**
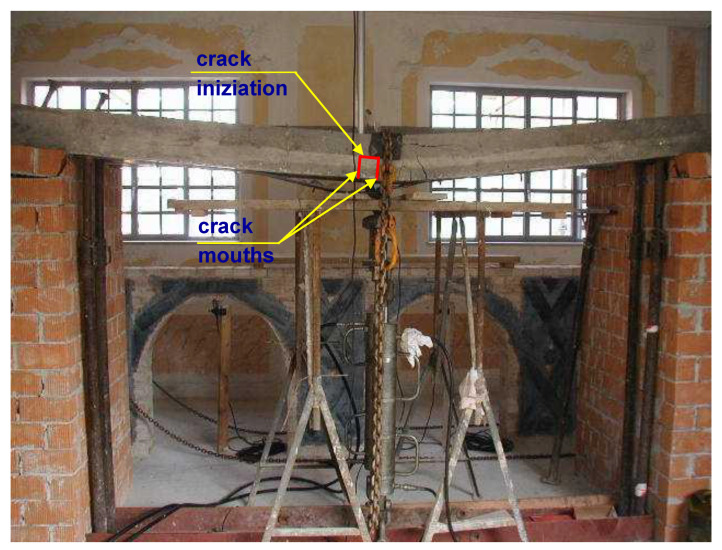
Experimental verification of the analytical model performed by means of four specimens tested up to collapse. The four beams were initially subjected to a two-point load, which produced vertical cracks around the midspan. Then, two beams were left unstrengthened, and two beams were strengthened in the fashion described in Section 4 (side-bonded reinforcement). Finally, a vertical force was applied to the midspan of each beam (pistons and actuators are shown in the picture) and was increased to failure. Once the side-bonded reinforcement debonded, the applied load caused the entire beam to collapse. The last event that led to the collapse of the beam was debonding of the longitudinal external FRP reinforcement applied on the lower side of the beam. Nevertheless, debonding of the flexural FRP reinforcement occurred after debonding of the shear CFRM reinforcement, when the load-carrying capacity was almost zero.

**Figure 8:**
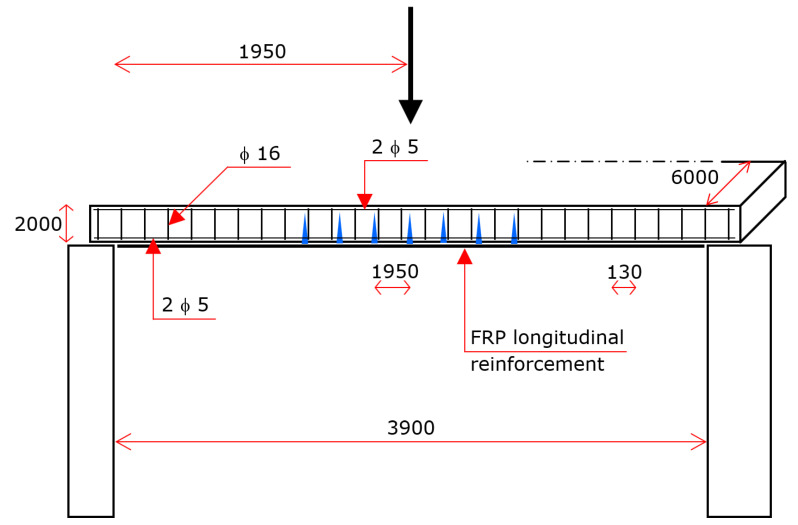
Diagram of the four identical RC beams that were tested. The figure shows the span, the dimensions of the cross-section, the longitudinal steel bars, the steel stirrups, and the externally bonded flexural reinforcement. The symbol φ denotes the diameter of a steel bars and a stirrup. Vertical cracks were produced around the midspan with a specific loading. The diagram also shows those cracks and their spacing. A vertical force (shown in the diagram) was applied to the midspan of each beam and was increased to failure.

The model is based on some assumptions, which, on one hand, are realistic, but on the other hand, suggest checking the actual predictive capacity of the model. Checking was accomplished by comparing the theoretical predictions to experimental results obtained from laboratory tests on four RC beams (Figure 7 and Figure 8). 

The four beams were identical, i.e., they had the same geometry and reinforcement and were made of the same concrete. The beams were rectangular. The height of the cross-section was 200 mm, and the width was 600 mm. Each specimen was simply supported over a span of 3900 mm (Figure 7 and Figure 8).

The steel reinforcement was designed in order to tie the cracks the less that was possible. Namely, the longitudinal steel bars that were placed allowed vertical cracks to attain large widths.

As explained below, the cracks were obtained applying to each beam two equal forces significantly greater than the theoretical forces that implied cracking of the concrete. Given that the greater the tension strain in the longitudinal steel reinforcement induced by a load, the greater the widths of the cracks that are produced by the load, the flexural steel reinforcement was reduced to an essential level. The longitudinal steel reinforcement consisted of bars with the minimum number and diameter necessary to make the cage and to cast the concrete, and the flexural reinforcement necessary to carry the loads was obtained with FRP external reinforcement.

On the contrary, the stirrups were made of steel bars with a particularly large diameter, in order to confirm that they play virtually no role in providing shear strength where cracks are vertical.

In geometrical terms, the steel reinforcement consisted of four longitudinal bars with diameters of 5 mm (two at the top and two at the bottom) and stirrups with diameter of 16 mm at the spacing of 130 mm (Figure 8).

The reinforcement that provided the beam with bending strength was obtained by epoxy-bonding longitudinal FRP strips onto the entire lower face of the beam (three layers).

As mentioned above, the beams underwent a preliminary procedure devoted to producing vertical cracks around the midspan. Every beam was subjected to two concentrated vertical forces, each one applied 0.975 m apart the midspan (i.e., the distance between the forces was 1.950 m). The magnitude of each force was 31.50 kN. It is of note that the theoretical value of the two forces that caused first cracking was 17.29 kN, and the collapse value was 36.50 kN.

Then, those two concentrated forces were taken away (the two-point load procedure was only the means to obtain RC beams with vertical cracks around the midspan). The result was the obtainment of four RC beams with quasi-vertical cracks for about 1950 mm around the midspan.

Then, two beams were strengthened in the fashion described in Section 4, while the other two beams were left unstrengthened.

Lastly, a concentrated load was applied at midspan of each beam, and it was increased up to failure. So, two beams were subjected to a collapse test without side-bonded reinforcement, and the other two beams were subjected to a collapse test with the side-bonded reinforcement described in Section 4.

The side-bonded reinforcement consisted of three layers of textile fabric per lateral face of the beam. Namely, *N* = 6.

The side-bonded reinforcement used for the tests consisted of an FRCM material. FRCM reinforcement was preferred to FRP reinforcement since debonding of FRCM consists of fibers that slip with respect to the matrix they are embedded into, whereas debonding of FRP consists of the rupture of a layer of concrete cover. For that reason, debonding of an FRCM reinforcement is more controllable in a laboratory test than debonding of an FRP reinforcement. The strain of the FRCM reinforcement at debonding was accurately measured, whereas the debonding strain of an FRP reinforcement could not have been measured so accurately. Moreover, the debonding strain of an FRCM reinforcement can be better predicted that debonding strain of an FRP reinforcement.

Those evaluations were confirmed by the experimental campaign. The FRCM debonding strain ε_Fd_ that had been predicted before executing the tests was in the range 2.75–2.85‰, while the average debonding strain that was measured in the two tests of the strengthened beams was 2.801‰. Moreover, the two experimental debonding strains (whose average value was 0.0028) differed marginally from one another. 

The characteristics of the external reinforcement as standalone component were: *t_F_* = 0.177 mm and *E_F_* = 244,000 N/mm^2^. 

The side-bonded external reinforcement was anchored onto the top and bottom faces of the beam. Accordingly, the side-bonded reinforcement guaranteed *L_F_* ≥ *L_eff_* both at the top and the bottom of each sheet. That condition is represented by η = 1 of Table 1.

The concrete compressive strength and tensile strength measured by cylinder tests were 41.6 N/mm^2^ and 4.2 N/mm^2^, respectively.

Ultimately, the one-point load experiments tested up to collapse four beams made of concrete with the ultimate stresses in tension and compression reported above, and with vertical cracks around the midspan. Two beams were tested without side-bonded reinforcement and two beams with the side-bonded reinforcement described above.

The initial two-point load procedure and the final one-point load test used high-tensile steel cables and actuators. The two-point load procedure applied the two forces with four prestressing tendons and four pistons (one high tensile steel cable and actuator per side and per point) to develop two constant vertical forces that caused the beam to crack by pure bending moment and the cracks to open widely. The one-point load test applied the force with two prestressing tendons and two pistons (one high tensile steel cable and actuator per side and per point) to develop an increasing vertical force up to collapse (Figure 7). 

Failure of the two beams without the side-bonded reinforcement was triggered by the concrete cantilever at midspan. Tension stresses on the built-in section reached concrete cracking stress.

Failure of the two beams with side-bonded reinforcement was triggered by debonding of the side-bonded external reinforcement at midspan crack. Tension strains in the fibers at the bottom of the lateral side reached the debonding strain.

The collapse loads of the tests of the two beams without side-bonded external reinforcement were 158.04 kN and 163.70 kN, respectively (Table 2).

Setting to zero the terms of Equation (35) that reproduce the contribution given by the side-bonded reinforcement, the equation provides *V_ud_* = 74.592 kN. Since β = 0.5, Equation (36) gives *P_ud_* = 149.184 kN (Table 2). 

The maximum difference between the two experimental failure loads and the theoretical failure load is 8.9%, which is marginal. A theoretical prediction lower than the experimental value is in agreement with the term that reproduces the contribution of the concrete cantilever. In fact, that term uses concrete tensile strength and not concrete flexural strength (the latter is 1.1–1.2 times greater than the former).

Not only do the tests confirm the predictive capacity of the model, but also that stirrups are ineffective when cracks are vertical. It is of note that the diameter of the stirrups was large. Despite this, the stirrups provided shear strength around the midspan with no contribution.

The collapse loads of the tests of the two beams with side-bonded external reinforcement were 240.40 kN and 235.18 kN, respectively (Table 2).

Plugging ε_Fd_ = 2.8‰ into Equation (35), the equation provides *V_ud_* = 108.377 N. Since β = 0.5, Equation (36) yields *P_ud_* = 216.754 kN (Table 2). 

The maximum difference between the two experimental failure loads and the predicted failure load is less than 11.0%, which is largely acceptable in order to validate the analytical model.

It can be concluded that the experimental results confirmed the reliability and accuracy of the analytical model.

## 9. Two Exemplificative Applications of the Model: Theoretical Predictions

The analytical model was applied to two virtual cases. Each case study consisted of an RC beam with vertical cracks around the midspan, strengthened in the fashion described in Section 4, and subjected to a concentrated load *P* at midspan (β = 0.5); for each of them, the ultimate value *P_ud_* of *P* is calculated using the model. The geometric and mechanical characteristics of the case studies are shown in Table 3. 

The ultimate values *P_ud_* of *P* provided by the model for the two case studies allow considerations to be drawn (Section 10).

Each beam was simply supported over a span of 5500 mm and had a tee cross-section (T-beam). Moreover, each RC beam was strengthened with an externally bonded FRP reinforcement attached onto both the side surfaces of the beam’s web, for the entire span (Table 3). The side-bonded reinforcement was made of textile fiber at ±45°, according to the sign of the shear (Section 4). The upper end of each sheet is at the bottom face of the flange. The lower end of each sheet is on the bottom face of the beam for a length related to *L_eff_*.

### 9.1. First Case Study

On assumptions (3) and (4), cracks exhibit the following pattern:

α = ξ = (2/3)·410 = 273 mm(37)

The concrete cover is 40 mm (Table 3). So, crack depth is equal to ξ’ = 273 + 40 = 313 mm. Spacing-to-depth ratio of cracks results to be 0.87, which is realistic.

The total thickness of the FRP reinforcement attached onto each side surface is *t_F-tot_* = 3·0.177 = 0.531 mm. The effective bond length turns out to be:

(38) $$\;L_{eff}\;=\;0.47\cdot \sqrt[{2}]{\;\frac{\;244,000\cdot 0.531}{\;1.14\;}}\;=\;158\;\mathrm{mm}$$

To obtain *L_F_* ≥ *L_eff_*, each sheet has to be anchored onto the whole width of the beam’s bottom side (*b* = 150 mm) and must then be bent onto the other side of the concrete section for no less than 8 mm.

The most efficient arrangement of the side-bonded FRP reinforcement needs μ ≥ ξ’. Namely, μ’ − 0.707·*L_eff_* ≥ 313 mm, in which *L_eff_* of Equation (38) has been used. The fabric sheet height μ’ should therefore satisfy the following relationship: μ’ ≥ 313 + 0.707·158 → μ’ ≥ 425 mm.

Unfortunately, the flange bounds the height of the sheet to: μ’ = 450 − 200 = 250 mm < 425 mm (as shown by Table 3, the flange is 200 mm thick). Given that the height of the web is 250 mm, the condition μ ≥ ξ’ is not obtainable.

Each fabric sheet is attached onto the entire height of the beam’s web to provide the side-bonded FRP reinforcement with the greatest possible value of μ’. So, μ = 250 − 0.707·158 = 138 mm. The crack depth surpasses the height of the side-bonded FRP reinforcement by 175 mm. This condition is accounted for by the coefficient η. The ratio μ/ξ’ is equal to 0.44. For μ/ξ’ = 0.44, Table 1 gives η = 0.65.

The full end-debonding strain is provided by Equation (7):

(39) $$\varepsilon_{\mathrm{Fd}}\;=\;0.35\cdot \frac{\;\sqrt[{4}]{13.2\cdot 1.14}\;}{\sqrt[{2}]{244,000\cdot 0.531}}\;=0.00192$$

The ultimate shear force is derived from Equation (35):

(40) $$\begin{array}{l} V_{ud}\;=\;0.148\cdot 150\cdot 410\cdot 1.14\;+\;0.314\cdot 0.00192\cdot 244,000\cdot 0.177\cdot 6\cdot 0.65\cdot 410\;\\ -\;0.280\cdot 0.00192\cdot 244,000\cdot 0.177\cdot 6\cdot 0.65\cdot \frac{\;410^{2}}{\;0.5\cdot 5500\;} \end{array}$$

from which

(41) $$V_{ud}\;=\;10,376.3\;+\;41,527.6-\;5521.0\;=\;10,376.3\;+\;36,006.6\;=\;46,382.9\;N$$

The concentrated load-carrying capacity is derived from Equation (36), plugging the result of Equation (41) and β = 0.5: *P_ud_* = 92765.8 N ≡ 92.8 kN.

The real role played by the side-bonded FRP reinforcement in strengthening this RC beams is represented by the ratio between the fraction of strength provided by the concrete cantilever and that provided by the FRP reinforcement:

(42) $$\frac{\;10,376.3\;}{36,006.6}=\;0.29\;=\;\frac{1}{\;3.5\;}$$

Ratio (42) confirms that the FRP reinforcement plays the major role in providing the beam with concentrated load-carrying capacity.

Without side-bonded FRP reinforcement, *P_ud_* would have been only 24.9 kN. It is of note that the above value of *P_ud_* (that without side-bonded FRP reinforcement) was derived using a concrete flexural strength to concrete tensile strength ratio equal to 1.20 (which is appropriate for this case).

Ultimately, the concentrated load-carrying capacity *P_ud_* with side-bonded FRP reinforcement is 3.7 times greater than without. In other words, the side-bonded FRP reinforcement has provided an increase in *P_ud_* of approximately 450%. 

Considering that both the concrete section and steel reinforcement of this beam are adequate for carrying uniform loads, this case study confirms that the strengthening technique is a viable method for the cases it is devoted to.

### 9.2. Second Case Study

Assumptions (3) and (4) allow the following condition to be establish:

α = ξ = (2/3)·650 = 433 mm(43)

According to Table 3, the concrete cover of this beam is 50 mm. The crack depth is therefore ξ’ = 433 + 50 = 483 mm, and the spacing-to-depth-ratio of the cracks is 0.90.

Equation (6) gives:

(44) $$L_{eff}=\;0.47\cdot \sqrt[{2}]{\;\frac{\;390,000\cdot 2\cdot 0.222}{\;1.01}\;}=195\;\mathrm{mm}$$

The flange thickness (Table 3) imposes the limit μ’ = 700 − 240 = 460 mm on the external reinforcement. On substituting the above value of μ’ and *L_eff_* of Equation (44) in the relevant expression, the effective depth of the side-bonded FRP reinforcement is: μ = 460 − 0.707·195 = 322 mm. 

The adopted side-bonded FRP reinforcement configuration implies that the crack is uncoated for 160 mm, i.e., μ/ξ’ = 0.67. That condition is accounted for by the coefficient η of Table 1, which is η = 0.87.

Equation (7) yields:

(45) $$\varepsilon_{\mathrm{Fd}}\;=0.35\cdot \frac{\;\sqrt[{4}]{11.0\cdot 1.01}\;}{\sqrt[{2}]{390,000\cdot 2\cdot 0.222}}\;=\;0.0015$$

The ultimate shear force *V_ud_* is derived from Equation (35):

(46) $$\begin{array}{l} V_{ud}\;=0.148\cdot 200\cdot 650\cdot 1.01\;+\;0.314\cdot 0.0015\cdot 390,000\cdot 0.222\cdot 4\cdot 0.87\cdot 650\;\\ \;-\;0.280\cdot 0.0015\cdot 390,000\cdot 0.222\cdot 4\cdot 0.87\cdot \;\frac{650^{2}}{\;0.5\cdot 5500\;} \end{array}$$

Equation (46) becomes:

(47) $$V_{ud}\;=\;19,432.4\;+\;94,430.6-\;19,903.1\;=\;19,432.4\;+\;74,527.4\;=\;93,959.8\;N$$

The concentrated load-carrying capacity is derived from Equation (36), plugging the result of Equation (47) and β = 0.5 into it: *P_ud_* = 187919.7 N ≡ 187.9 kN

The actual role played by the side-bonded FRP reinforcement in strengthening this RC beams is represented by the ratio between the fraction of strength provided by the concrete cantilever and the fraction provided by the FRP reinforcement:

(48) $$\frac{\;19,432.4\;}{74,527.4}=\;0.26\;=\;\frac{1}{\;3.8\;}$$

Ratio (48) confirms that the FRP reinforcement plays a major role in providing the beam with concentrated load-carrying capacity.

Without side-bonded FRP reinforcement, *P_ud_* would have been only 46.6 kN. Again, *P_ud_* without side-bonded FRP reinforcement was derived using a ratio between flexural strength and tensile strength of concrete equal to 1.20.

Ultimately, the concentrated load-carrying capacity *P_ud_* with side-bonded FRP reinforcement is 4.0 times greater than without. In other words, the side-bonded FRP reinforcement has provided an increase in *P_ud_* of approximately 400%. 

Considering that both the concrete section and steel reinforcement of this beam are adequate to carry uniform loads, even this case study confirms that the technique is a viable method for the cases it is devoted to strengthening.

## 10. Interpretation of the Results and Discussion

The case studies of the preceding section are emblematic for beams of building, road, rail, or waterway structures. As such, those case studies allow interpretation and general conclusions to be drawn. 

Concrete sections and steel reinforcement arrangements that are adequate for carrying uniform loads conversely are inadequate to carry concentrated loads applied around the midspan. So, if the applied load has to be changed from distributed along the span to concentrated around the midspan, the beam must be strengthened.

Side-bonded composite reinforcement with fibers at ±45° (and with the arrangement of Section 4) can drastically increase the concentrated load-carrying capacity of an RC beam.

The side-bonded fiber composite reinforcement prevents dowel action and aggregate interlock from reaching appreciable levels, as well as the concrete cantilever from exhibiting substantial plasticity. Nevertheless, the contributions of dowel action and aggregate interlock that are missed are irrelevant, since around the midspan (where cracks and concrete cantilevers are vertical), dowel action and aggregate interlock boost shear strength no more than marginally. Moreover, the cantilever reaches the concrete tensile strength. So, the resisting bending moment of the concrete cantilever is only slightly lower than the bending moment it could reach due to the plasticity it could display (concrete flexural strength).

On one hand, the high ratio between the concentrated load-carrying capacity of the strengthened beam and that of the unstrengthened beam obtained for the case studies is due to the fact that the denominator is small. In fact, the shear-carrying capacity around the midspan is small, and so is the concentrated load-carrying capacity.

On the other hand, however, the externally bonded reinforcement makes concentrated load-carrying capacity increase significantly, not only relative to the value it had in the unstrengthened condition, but also in absolute terms. As a matter of fact, the concentrated load-carrying capacity that can be reached allows an RC beam to carry not only the same resultant force it carried before though now in a concentrated fashion rather than in the preceding distributed fashion, but also substantially higher concentrated forces.

The above evaluation underlies a general reflection. Externally bonded fiber composite materials can increase the distributed load-carrying capacity, bending-carrying capacity, shear-carrying capacity at the beam’s ends, axial-carrying capacity of a column, and dissipation capacity no more than moderately. On the contrary, they can increase the concentrated load-carrying capacity substantially.

The sensitivity analysis has shown that increasing the full end debonding strain of the side-bonded FRP reinforcement would allow the concentrated load-carrying capacity to increase in proportion. That relationship between debonding strain and capacity is, however, typical for any FRP reinforcement.

Full end debonding strain can be increased by anchoring the external reinforcement with stud shear connectors [56,57,58,72] or by making the resin penetrate the concrete to a greater depth [58]. The former technique can be performed by piercing the concrete under the guidance of a magnetometer survey (pacometer), which indicates the portions of concrete without steel, so as to know the zones that can be drilled. The latter technique can be performed by applying the externally bonded reinforcement with a vacuum technique [58].

The model shows that the relationship between *V_ud_* and *d* is not far from linear. That result may drive the design of the reinforcement.

## 11. Conclusions: Review of the Implications of What Presented

The conventional shear capacity models of RC beams have been used for many decades with satisfactory results, which proves their reliability (the first and classical shear model dates back to 1899). Those models have, however, mainly been used for new structures, and their use for existing structures started only few decades ago. Moreover, in the case of existing RC beams, the shear capacity models have been mainly applied to predict the increase in ultimate load factor that a certain strengthening technique allows the beam to obtain.

On the contrary, shear capacity models of RC beams have not been tested on beams whose load distribution is to be changed (or has been changed) from distributed to concentrated. 

This paper has proven that, in those cases, the conventional shear capacity models of RC beams fail. Not only are those models unsuccessful, but also conventional strengthening techniques fail in those cases, including the most common one—namely, externally bonded fiber composite reinforcement.

Aiming at filling those gaps, the paper has presented a strengthening technique together with the relevant predictive model. The strengthening technique consists of side-bonded reinforcement with fibers at 45°. The model consists of an analytical formulation that predicts the concentrated load-carrying capacity of the RC beams in the strengthened state.

The assumptions that the model is based on relate to crack spacing and crack depth. Those assumptions are in accordance with real observation, so they are realistic. Nonetheless, the expansion of the shear capacity provided by the model in a Taylor series about the assumed conditions (ξ = *d*/1.5 and α = ξ) proves that the error would be moderate even if ξ and α were substantially far from the assumed values. 

The last significant modeling assumption is that, at debonding failure, stresses acting on the built-in end of the concrete cantilever have a bi-triangular distribution, and that the maximum stress of the distribution is equal to concrete tensile strength. Research activity has proven that, at debonding failure of external reinforcement, the stress distribution is elasto-plastic, which means that the assumed stress profile is both realistic and conservative.

No further substantial assumptions were made. The proposed model hence provides reliable predictions. That statement has also been proven by the experimental campaign that was performed in order to check the model, which confirmed that the theoretical predictions are in agreement with the experimental results. 

The case studies have proven that the proposed externally bonded reinforcement can increase the concentrated load-carrying capacity of an RC beam substantially, which is a result that externally bonded reinforcement cannot guarantee when used to increase other types of capacities of RC structures.

The strengthening technique is also applicable to structures for which the application point of the concentrated load is not fixed but can vary its position. In that case, nevertheless, the fabric sheets have to be bi-directional—namely, fibers both at +45° and −45° on each side of the web.

## Figures and Tables

**Table 1:** Approximate values of η to use in lieu of the analytical exact values. The approximate η are expressed as a function of the μ/ξ’ ratio.

μ/ξ’ > 0.80	0.80 > μ/ξ’ > 0.65	0.65 > μ/ξ’ > 0.50	0.50 > μ/ξ’ > 0.35	0.35 > μ/ξ’ > 0.20
η = 1	η = 0.87	η = 0.77	η = 0.65	η = 0.45

**Table 2:** Comparison between the experimental results from the four collapse tests (failure concentrated force of each test and observed failure mode) and the theoretical results from the analytical model (concentrated load-carrying capacity *P_ud_* and theoretical failure mode).

	Unstrengthened Beams	Strengthened Beams
	Collapse load of the first test	Collapse load of the first test
Experimental results	158.04 kN (failure of the cantilever)	240.40 kN (debonding)
from the tests	Collapse load of the second test	Collapse load of the second test
	163.70 kN (failure of the cantilever)	235.18 kN (debonding)
Experimental results	Concentrated load-carrying capacity	Collapse load
from the model	149.184 kN (failure of the cantilever)	216.754 (debonding)

**Table 3:** Geometric and mechanical characteristics of the two case studies. Symbols used for the tee cross-sections (T-beams): overall depth *H*, width of the web *b*, effective depth *d*, concrete cover *t*, and thickness of the flange *s*. The width of the flange plays no role, so it is not provided (any width is possible). Symbols used for the FRP reinforcement: thickness of each layer of reinforcement *t_F_*, total number of layers *N*, and elastic modulus *E_F_*. Symbols used for the concrete: cylinder compressive strength *f_cd_* and cylinder tensile strength *f_ctd_*.

*H* mm	*b* mm	*d* mm	*t* mm	*s* mm	*t_F_* mm	*N*	*f_cd_* N/mm^2^	*f_ctd_* N/mm^2^	*E_F_* N/mm^2^
450	150	410	40	200	0.177	3 + 3	13.2	1.14	244,000
700	200	650	50	240	0.222	2 + 2	11.0	1.01	390,000
